# *SYDE1* Acts as an Oncogene in Glioma and has Diagnostic and Prognostic Values

**DOI:** 10.3389/fmolb.2021.714203

**Published:** 2021-10-14

**Authors:** Zhenyuan Han, Xiaodong Zhuang, Biao Yang, Lihui Jin, Pengjie Hong, Junqing Xue, Shunjie Chen, Zhen Tian

**Affiliations:** ^1^ Department of Oral and Maxillofacial Surgery, Peking University School and Hospital of Stomatology, Beijing, China; ^2^ Department of Oral Pathology, Shanghai Ninth People’s Hospital, Shanghai Jiao Tong University School of Medicine, Shanghai, China; ^3^ National Clinical Research Center for Oral Diseases, Shanghai, China; ^4^ Department of Stomatology, Affiliated Fuzhou First Hospital of Fujian Medical University, Fuzhou, China; ^5^ Department of Neurosurgery, Huashan Hospital of Fudan University, Shanghai, China; ^6^ Department of Pediatric Cardiology, Xinhua Hospital, Affiliated to Shanghai Jiao Tong University School of Medicine, Shanghai, China; ^7^ Department of Nephrology, Shanghai Fourth People’s Hospital Affiliated to Tongji Universtiy, Shanghai, China

**Keywords:** SYDE1, glioma, unfavorable clinicopathological factors, prognosis, diagnosis

## Abstract

**Objectives:** Gliomas remain one of serious public health problems worldwide which demand further and deeper investigation. The aim of this study was to explore the association between synapse defective protein 1 homolog 1 (*SYDE1*) and gliomas *via* public database analysis and *in vitro* validation to determine the potential diagnostic and prognostic values.

**Methods and Results:** Compared with healthy brain tissues, there was a significant increase in *SYDE1* expression in glioma tissues. Additionally, *SYDE1* exhibited higher expression levels in glioma patients with unfavorable clinicopathological factors. *In vitro* knockdown of *SYDE1* in glioma cell lines A172 inhibited their migrative and invasive ability but not the proliferative ability. GO and KEGG pathway analysis of the top 100 genes coexpressed with *SYDE1* showed enrichments of tumor-associated terms. Further bioinformatic analysis revealed that the *SNHG16/hsa-miR-520e/SYDE1* axis might be involved in glioma development.

**Conclusions:** *SYDE1* is expressed at higher levels in gliomas than in healthy brains, and can promote metastasis and invasion but not proliferation of gliomas. Furthermore, *SYDE1* has values in the diagnosis and prognosis prediction of gliomas.

## Introduction

Gliomas are one of the most common invasive malignancies in the central nervous system, accounting for approximately 51.4% of all primary brain tumors (Xue et al., 2017). In particular, gliomas constitute the majority of primary brain tumors in adults (Goodenberger and Jenkins, 2012). The current therapeutic approach for gliomas, however, is limited to complete surgical resection combined with radiotherapy and alkylating chemotherapy (Louis et al., 2016). Of note, since oncogenes are capable of sustaining tumor growth and conventional treatment does not take into account special parameters of various subtypes, the expectation of novel treatments is still grave for the general majority of patients (Arko et al., 2010; Gilbert et al., 2013; Kamiya-Matsuoka and Gilbert, 2015).

According to the 2007 WHO classification of central nervous system tumors, gliomas can be divided into low-grade glioma (LGG, grade I and II) and high-grade glioma (HGG, grade III and IV) (Louis et al., 2007). This classification is mainly based on the clinical and histopathological features of the glioma. The latest version of the WHO glioma category released in 2016 has included the evaluation of molecular characteristics, such as isocitrate dehydrogenase (IDH) mutations, chromosome 1p/19q codeletion and RELA fusion positivity (Louis et al., 2016). IDH enzymes catalyze the oxidative decarboxylation of isocitrate and are essential in maintaining cellular homoeostasis. IDH mutations have been reported by Yan et al. to occur in >80% of HGG cases (Yan et al., 2009). Chromosome 1p/19q codeletion is recognized as another hallmark of gliomas, which presents in approximately 60–90% of oligodendrogliomas and 30–50% of oligoastrocytomas (Hofer and Lassman, 2010). Of note, 1p/19q codeletion is associated with a better response to radiotherapy and chemotherapy and can predict longer progression-free and overall survival (Dunbar, 2009). Given that IDH mutations and 1p/19q codeletions occur mostly in different subtypes of gliomas, the 2016 edition of the WHO glioma classification faces great challenges in clinical practice. Therefore, it is of great necessity to acquire an in-depth understanding of gliomas and identify other causal genes.

Synapse defective protein 1 homolog 1 (*SYDE1*) is a 79-kDa Rho GTPase-activating protein that is encoded by the *SYDE1* gene located at chromosome 19p13.12. *SYDE1* is highly expressed in the placenta, bone marrow and brain and is involved in the positive regulation of placental trophoblast cell migration and bone marrow cell differentiation (Lo et al., 2017). Moreover, *SYDE1* has been revealed to be differentially expressed between cervical cancer and normal controls and is recognized as a potential causal gene related to cervical cancer (Zhang et al., 2021). Buchner et al. deciphered the role of *SYDE1* in renal cell carcinoma (RCC) via analysis of metastatic RCC mRNA expression profiles, which revealed that *SYDE1* could discriminate well between RCC patients with favorable prognosis or poor prognosis (Buchner et al., 2010). Given the high expression level of *SYDE1* in brain tissues, it is of great necessity to determine the function of *SYDE1* in the pathogenesis of cerebral tumors, especially gliomas.

In this study, we utilized publicly available data from the Oncomine, GEPIA2 and Human Protein Atlas (HPA) databases to examine SYDE1 expression in glioma tissues and normal control tissues, and we identified an increased level of SYDE1 in gliomas. Furthermore, *SYDE1* expression was higher in HGGs than in LGGs, as revealed by bioinformatic analysis of the Gene Expression Omnibus (GEO), The Cancer Genome Atlas (TCGA) and Chinese Glioma Genome Atlas (CGGA) datasets. Immunohistochemistry (IHC) staining of SYDE1 human glioma samples from grades I to IV draws the same conclusion. Then, we performed RNAi-mediated *SYDE1* knockdown in glioma cell lines *in vitro*, which revealed that *SYDE1* knockdown abolished the migration and invasion of glioma cells. To decipher the mechanisms of *SYDE1* in glioma pathogenesis, we further constructed a coexpression network of genes in gliomas from the cBioPortal database whose Spearman’s correlation index values with *SYDE1* rank in the top 100 and found that these genes were enriched in tumor-associated GO terms.

## Materials and Methods

### Application of Public Web-Based Databases

Gene expression profiling interactive analysis 2 (GEPIA2; http://gepia.cancer-pku.cn/index.html) is a Chinese online tool for bioinformatics and visualized analysis based on the TCGA and GTEx databases. It is widely used because of its convenient operation and high efficiency although users do not have access to the primary data (Tang et al., 2019). The present study used survival analysis of *SYDE1*, hsa-miR-136, hsa-miR-302a, hsa-miR-424, hsa-miR-497, hsa-miR-529e, FGD5-AS1, MIR17HG, and SNHG16 to explore the relationships between *SYDE1* expression and glioma prognosis and pathology.

To explore the expression level of *SYDE1* in CNS/brain tumors (especially gliomas) and normal brain tissues, Oncomine (https://www.oncomine.org) was utilized to analyze the web-based data on the expression of *SYDE1* in different types of gliomas and corresponding normal samples (Rhodes et al., 2004). This study compared the pattern of the expression of *SYDE1* in four major types of gliomas, including GBMs, anaplastic astrocytomas, diffuse astrocytomas, and oligodendrogliomas, with the inclusion threshold described as a fold-change (FC) > 1.5, *p* < 0.01, and gene rank = all.

The HPA (https://www.proteinatlas.org) is a widely used online protein analysis database that contains 26,000 human proteins, and it was used to develop immunoassay technologies to analyze the expression levels of proteins in cell lines, human normal tissues and tumor tissues (Uhlén et al., 2015). The present study assessed the translational expression levels of *SYDE1* in gliomas and normal brain tissues.

The top 100 coexpressed genes of *SYDE1* were acquired from the cBioPortal database (http://www.cbioportal.org/), and were visualized *via* Cytoscape (https://cytoscape.org/) (Cerami et al., 2012; Gao et al., 2013). The mRNA/miRNA/lncRNA interactions were forecasted using starBase 3.0 (http://starbase.sysu.edu.cn/), which includes seven supported algorithms (PITA, miRmap, microT, miRanda, PicTar, RNA22, and TargetScan) (Li et al., 2014). Target miRNAs predicted by at least six algorithms were selected for further functional analysis.

### Data Obtaining and Preprocessing

To evaluate the relations between *SYDE1* expression and cancer prognosis and other clinicopathological characteristics, *SYDE1* mRNA expression profiles in glioma samples and normal samples were downloaded from TCGA, CGGA and GEO. These mRNA profiles were quality controlled1 using RSeQC, and were normalized using the trimmed mean of M-values. Within the TCGA database, we subgrouped the glioma samples into three major cohorts, glioma, LGG, and GBM samples, which were named TCGA_glioma, TCGA_LGG, and TCGA_GBM, respectively. For the Chinese cohorts, *SYDE1* mRNA expression and its corresponding clinicopathological characteristics were downloaded from CGGA (http://www.cgga.org.cn/), and these cohorts included three datasets, mRNA-array_301, mRNAseq_325, and mRNAseq_693 datasets, with 301, 325, and 693 glioma tissue samples, respectively. Several microarray datasets from the GEO database were also selected for our present study, including GSE4271 (generated from GPL96), GSE4290 (generated from GPL570), GSE4412 (generated from GPL96), GSE68848 (generated from GPL570) and GSE13041 (generated from GPL96, GPL570 or GPL8300). The prognostic values between miRNAs and lncRNAs were assessed by CGGA microRNA-array_198 and CGGA mRNA-seq 325, respectively.

### Gene Set Enrichment Analysis

698 glioma cases were divided into two expression level groups based on the median expression value of *SYDE1*. GSEA was then conducted to identify hallmark gene sets that were enriched in the gene rank in the two groups. The h.all.v7.4.symbols.gmt in the Molecular Signatures Database (MSigDB) was selected in GSEA version 4.1 to annotate gene sets. The cutoff criteria were set to nominal *p* < 0.05, normalized enrichment scores (NES) > 1.0 and false discovery rate (FDR) q > 0.25. Finally, hallmark gene sets with significant enrichment were chosen, and gene set enrichment plots were made.

### Tissue Samples

This study included 40 human glioma samples (grade I: 5, grade II: 10, grade III: 10, and grade IV: 15) and five normal brain samples. Specifically, human glioma samples were paraffin-embedded and retrieved from Shanghai Ninth People’s Hospital. Normal brain samples were obtained from patients diagnosed with cerebral trauma surgery but without other brain diseases, which were collected during surgery after informed consent was obtained from patients who needed brain trauma surgery. The clinicopathological characteristics of 45 glioma and control cases is summarized in Table 1. This study was approved by the ethical committee of Shanghai Ninth People’s Hospital.

**TABLE 1:** Clinicopathological characteristics of patient samples and expression of *SYDE1* in glioma and normal tissues.

Characteristics	Category	Number of cases (%)
Age (years)	<45	20 (44.4%)
	≥45	25 (55.6%)
Gender	Female	24 (53.3%)
	Male	21 (46.7%)
WHO grade	Normal	5 (11.1%)
	I	5 (11.1%)
	II	10 (22.2%)
	III	10 (22.2%)
	IV	15 (33.3%)
Histology	Normal	5 (11.1%)
	Pilocytic astrocytoma	5 (11.1%)
	Oligoastrocytoma	3 (6.7%)
	Astrocytoma	7 (15.6%)
	Anaplastic astrocytoma	9 (20.0%)
	Anaplastic oligodendroglioma	1 (2.2%)
	Glioblastoma	15 (33.3%)
Expression of *SYDE1*	Low expression	22 (48.8%)
	High expression	23 (51.1%)

All mouse strains used in this study were on the C57BL/6 background and were approved by the ethical committee of Shanghai Ninth People’s Hospital. Cerebrums were dissected and dissolved in TRIzol for RNA extraction.

### Immunohistochemistry

Immunohistochemistry (IHC) was performed on paraffin-embedded human glioma and normal brain tissues collected from Shanghai Ninth People’s Hospital. The sections were deparaffinized in a xylene gradient and rehydrated in an ethanol gradient. Antigen retrieval was performed in sodium citrate buffer (10 mM sodium citrate pH 6.0) at 100 C for 20 min. Then, endogenous peroxidase was deactivated by applying 3% H_2_O_2_ in methanol. IHC of SYDE1 was performed by the Dako EnvisionTM method. Briefly, the sections (3 μm) were sequentially incubated with the anti-SYDE1 antibody (NBP1-89350, Novus Biologicals) and the HRP-conjugated secondary antibody (ab6721, Abcam). Then, the sections were color-developed with a DAB Immunohistochemistry Color Development Kit (E670033, Sangon Biotech) and counterstained with hematoxylin.

A Leica LF200 microscope was used to image the stained sections. The intensity of SYDE1 signals was scored as negative (0), weak (1) or strong (2). The staining extent of SYDE1 was evaluated according to the immunoreactive tumor cell percentage, which was scored as I (0%, score = 0), II (1–5%, score = 1), III (6–25%, score = 2), IV (26–75%, score = 3) and V (76–100%, score = 4). SYDE1 signals (ranging from 0 to 8) were calculated by multiplying the intensity score with the staining extent score and were classified into low (0–4) or high (5–8) groups for Fisher’s exact tests.

### Cell Culture

Human astroglial A172 cells and mouse glioma GL261 cells were gifts from the Shanghai Institutes for Biological Sciences. All cell lines were confirmed to be free of microorganism contamination. Glioma cell lines were grown in DMEM (Gibco) supplemented with 10% fetal bovine serum (FBS) and 1% (100×) streptomycin/penicillin.

### RNA Extraction and qPCR

Total RNA was extracted from cerebrums or cultured glioma cell lines using TRIzol and then reverse transcribed into cDNA using a Superscript II reverse transcriptase Kit. qPCR was carried out using Power SYBR Green PCR Master Mix on the Applied Biosystems 7900HT Fast Real-Time PCR System. qPCR primers used to detect the expression levels of *SYDE1* mRNA in human or mouse tissues were as follows: human forward 5′-CAT​CAT​CCA​GAA​GTG​CGT​TG-3′ and reverse: 5′-AAT​CCT​TGA​GGA​TGC​CAG​TG-3′; mouse forward: 5′-CCT​ACC​AAA​ACC​TCC​CGT​ACC-3′ and reverse: 5′-GGG​GCG​GTC​CTC​TCT​CTA​TC-3′. The relative expression of each gene was normalized to ACTB and calculated using the 2^−ΔΔCT^ method.

### Transfection of siRNA

The siRNA targeting human *SYDE1* (siSYDE1) and the non-targeting control siRNA (siNC) were obtained from Shanghai Zorin Biological Technology. The sequence of siSYDE1 was 5′-UAGUGGGACUGUACCGUCUUUdTdT-3′ (sense) and 5′-AAAGACGGUACAGUCCCACUAdTdT-3′ (antisense). The sequence of siNC was 5′-UUCUCCGAACGUGUCACGUdTdT-3′ (sense) and 5′-ACGUGACACGUUCGGAGAAdTdT-3′ (antisense). LipoRNAiMAX transfection reagent was applied to deliver the siSYDE1 or siNC into glioma cell line A172. Transfection was performed according to the manufacturer’s instructions when the cell monolayer reached 70–80% confluency.

### Cell Proliferation Assay, Wound Scratch Assay and Transwell Assay

A172 cell lines were used for cell proliferation, wound scratch and Transwell assays. SYDE1-knockdown and control cells were seeded into 96-well plates (1,500 cells/well) for the cell proliferation assay. Cell Counting Kit-8 (CCK-8; Beyotime, C0038) was used to measure cell proliferation according to the manufacturer’s instructions. Briefly, 10 μl of CCK-8 reagent was added to each well at the indicated detection times. The plates were incubated at 37°C for 1 h and then measured in a microplate reader at a wavelength of 450 nm to determine the optical density.

For the wound scratch assay, SYDE1-knockdown and control cells were placed into 6-well plates (1,500 cells/well). After confluence, a sterile pipette tip was used to scratch a straight line in the glioma monolayer cell. Images were recorded at 0 and 48 h after scratching, and ImageJ software was used to measure the scratch area.

Transwell assays were carried out using a 6-well culture insert (GIBCO, 140,640) according to the manufacturer’s instructions. SYDE1-knockdown and control cells were seeded in each well (20,000 cells/well). After culturing at 37°C for 24 h, the culture insert was removed. Photographs were recorded at 0 and 48 h after culture-insert removal to investigate and analyze the healed wound area.

### Statistical Analysis

Statistical details are available in the figure legends. Unpaired/paired Student’s t-test (two-tailed) was used to analyze differences between two groups. One-way ANOVA was applied to analyze differences among three or more groups. Fisher’s exact test was used for the IHC quantification of *SYDE1* expression in glioma patient samples and normal brain tissues. All data are presented as the mean ± standard deviation (SD) if not further implicated and considered statistically significant with a *p* value <0.05. All analyses were conducted in GraphPad Prism 7 or Microsoft Excel.

**FIGURE 1:**
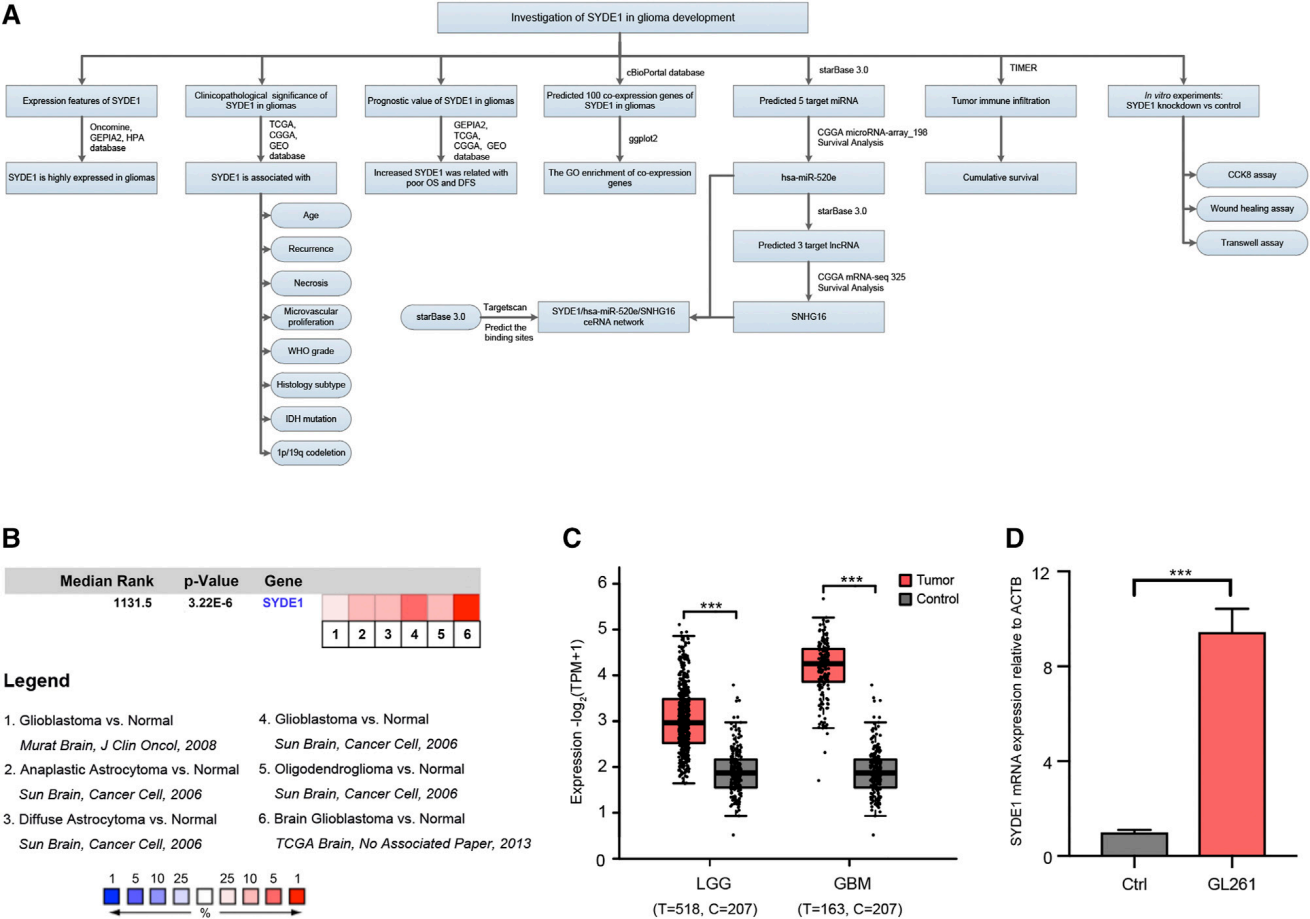
The comparison of *SYDE1* expression between normal brain and glioma tissues. **(A)** A schematic overview of different steps taken to examine the association between *SYDE1* and glioma. **(B)** *SYDE1* expression in normal human brains and different glioma subtypes. The rank for a gene is the median rank for that gene across each of the analysis and the p.value for a gene is its p.value for the median-ranked analysis. **(C)** *SYDE1* expression in normal human brains, LGGs and GBMs. **(D)** *SYDE1* expression in normal mouse brains and mouse glioma GL261 cells. ***, p.value <0.001; Ctrl, normal C57BL6 mouse brain.

For glioma survival analysis, Kaplan-Meier analysis and Cox regression analysis were combined to analyze the prognostic value of SYDE1. Kaplan–Meier survival plots were used to evaluate patient OS and DFS. Log-rank tests were used to determine significant differences between two groups. The overall design of our study is presented in the flow chart in Figure 1A.

## Results

### The Expression of *SYDE1* Significantly Correlated With the Clinical Features of Glioma Tissues

To explore expression levels of *SYDE1* in gliomas and their relation to clinical features and molecular subtypes, *SYDE1* expression levels in pairs of gliomas and adjacent normal samples were compared using the Oncomine and GEPIA2 databases. We identified four analyses of upregulated *SYDE1* between brain or CNS cancer and adjacent normal tissues in the Oncomine database that met the established threshold for |log_2_FC| >1.5, *p* < 0.01 and gene rank = all (Figure 1B). The GEPIA2 databases indicated that *SYDE1* was expressed at higher levels in glioma subclasses LGG and GBMs than in the corresponding normal tissues (*p* < 0.05, Figure 1C). Both databases support the hypothesis that *SYDE1* is upregulated in CNS tumors, such as gliomas, versus normal tissues. On the other hand, *SYDE1* expression is higher in mouse glioma GL261 cells than normal C57BL/6 brains (Figure 1D).

We assessed the relationship between the clinicopathological parameters and *SYDE1* expression. As shown in Supplementary Tables S1–S6, *SYDE1* expression significantly correlated with age in CGGA mRNA-array_325, CGGA mRNA-array_693, GSE4271, and TCGA_glioma (*p* < 0.05). For the histological subclasses, *SYDE1* expression was highly associated with this category in the CGGA mRNA-array_301, CGGA mRNA-array_325, CGGA mRNA-array_693, TCGA_LGG, and TCGA_glioma databases (*p* < 0.05). The analytical statistics also suggested that *SYDE1* was involved in WHO grade (*p* < 0.05), and the updated WHO classification-related diagnostic molecular characteristics IDH mutation and 1p19q_codeletion were also associated with *SYDE1* expression in CGGA mRNA-array_325 and CGGA mRNA-array_693 (*p* < 0.001). Our data also revealed that *SYDE1* was significantly associated with the PRS type in CGGA mRNA-array_325 and CGGA mRNA-array_693 (*p* < 0.05) and chemotherapy and radiotherapy in CGGA mRNA-array_325 and CGGA mRNA-array_693 (*p* < 0.001). Notably, the *SYDE1* marker was also related to microvascular proliferation and necrosis in GSE4271 (*p* < 0.05). Overall, these findings confirm that *SYDE1* expression is associated with different clinical outcomes and previous diagnostic biomarkers, which may benefit the diagnosis and treatment of gliomas.

### Increased *SYDE1* Expression Was Positively Related to Older Age, Recurrence, Necrosis, and Microvascular Proliferation in Gliomas

**FIGURE 2:**
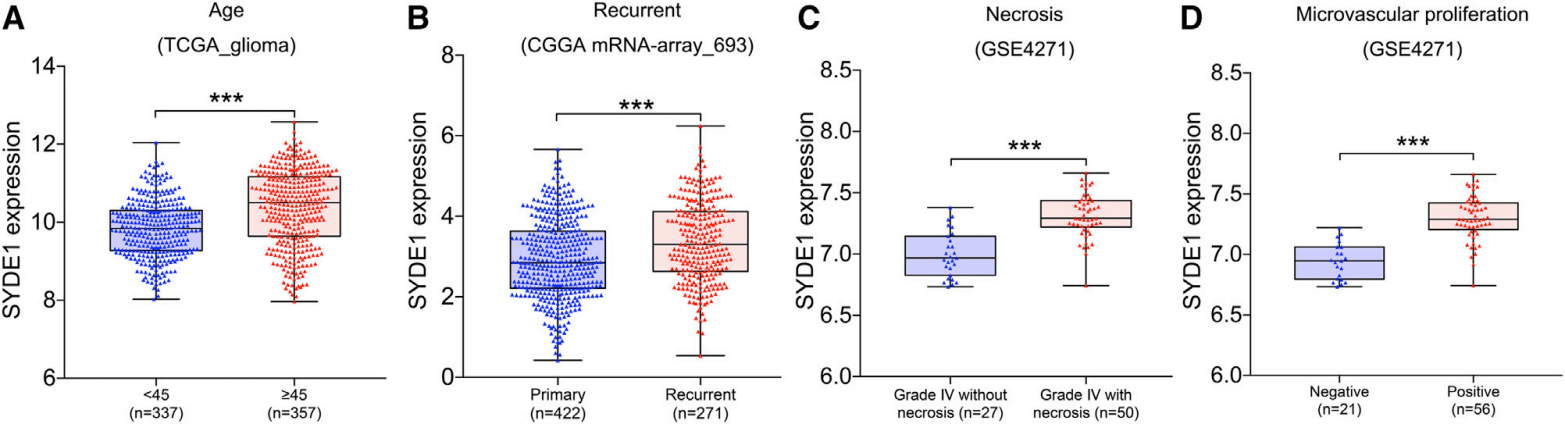
The association between *SYDE1* expression and patient age, tumor recurrence, tumor necrosis or tumor microvascular proliferation. **(A)** The association between *SYDE1* expression and patient age. **(B)** The association between *SYDE1* expression and glioma recurrence. **(C)** The association between *SYDE1* expression and glioma necrosis. **(D)** The association between *SYDE1* expression and glioma microvascular proliferation. *, p.value <0.05; ***, p.value <0.001.

To ensure the functionality of *SYDE1* in glioma development and clinical parameters, the *SYDE1* gene expression levels were divided into two groups according to age, with a cutoff of 45 years. There was a distinguished difference between the two age groups with differential *SYDE1* expression. Notably, the expression of *SYDE1* in older patients aged ≥45 years was higher than that in patients aged <45 years in the CGGA mRNA-array_301 (*p* < 0.05, Supplementary Figure S1A), CGGA mRNA-array_325 (*p* < 0.05, Supplementary Figure S1B), GSE4271 (*p* < 0.05, Supplementary Figure S1C), and TCGA_glioma datasets (*p* < 0.05, Figure 2A). There was no significant difference between the glioma patient groups regarding sex or race. *SYDE1* was chosen for further analysis because it closely correlated with the clinical features and because there were a small number of reports of its involvement in tumorigenesis. We also noted that the expression levels of *SYDE1* were significantly upregulated in the recurrent groups compared to the primary groups in CGGA mRNA-array_325 (*p* < 0.05, Supplementary Figure S1D) and CGGA mRNA-array_693 (*p* < 0.05, Figure 2B). One of the GBM diagnostic criteria, microvascular proliferation, and necrosis, was also related to *SYDE1* expression. Higher microvascular proliferation and grade IV necrosis occurred with higher *SYDE1* expression in gliomas (*p* < 0.05, Figures 2C,D). We evaluated these findings in the GSE4271 dataset.

### High Expression of *SYDE1* Indicated Higher WHO Grade and More Malignant Histological Subtypes in Gliomas

**FIGURE 3:**
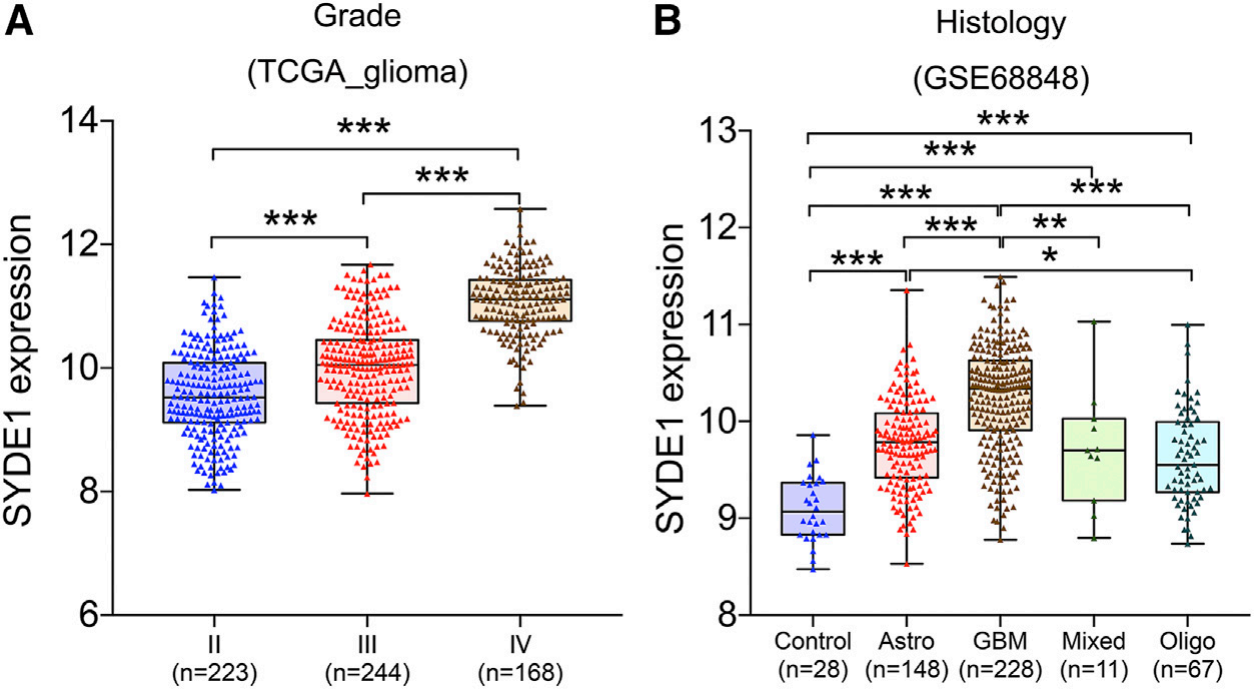
The association between *SYDE1* expression and glioma grade or histology. **(A)** The association between *SYDE1* expression and glioma grades. **(B)** The association between *SYDE1* expression and glioma histology. *, p.value <0.05; **, p.value <0.01; ***, p.value <0.001.

**FIGURE 4:**
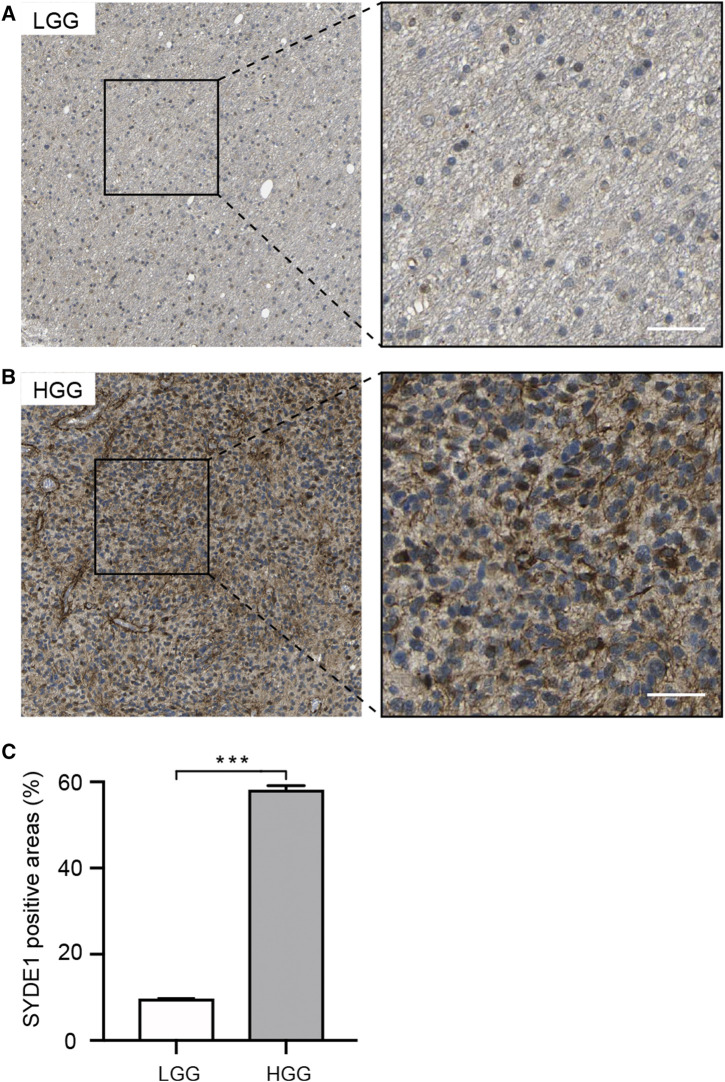
Immunohistochemical analyses of SYDE1 expression in LGG and HGG (The HPA database). **(A)** Weak SYDE1 staining in LGG. **(B)** Strong SYDE1 staining in HGG. **(C)** Quantification of SYDE1 positive areas in LGG and HGG. ***, p.value <0.001; Scale bars = 50 μm.

**FIGURE 5:**
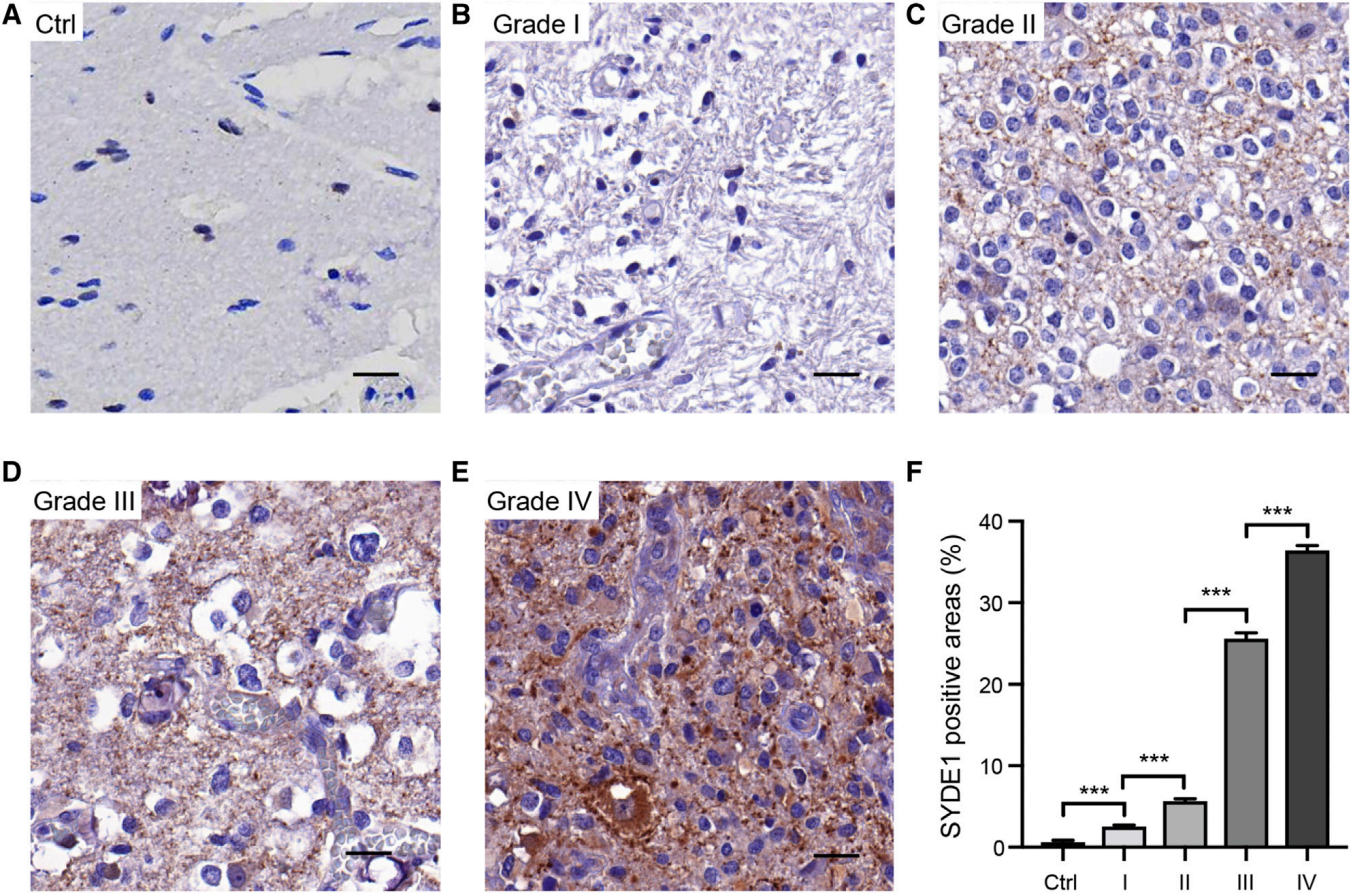
Immunohistochemical analyses of SYDE1 expression in resection specimens of normal human brain and grade I to IV glioma. **(A)** Negative SYDE1 staining in normal human brain. **(B–C)** Weak SYDE1 staining in grade I glioma **(B)** and grade II glioma **(C)**. **(D–E)** Strong SYDE1 staining in grade III glioma **(D)** and grade IV glioma **(E)**. **(F)** Quantification of SYDE1 positive areas at A-E. ***, p.value <0.001; Scale bars = 25 μm.

We first observed that *SYDE1* was highly expressed in gliomas, and an important, but unsolved, question is whether increased expression of *SYDE1* is related to increasing glioma grade. The mRNA expression patterns of *SYDE1* in different grades were further evaluated in the GEO, CGGA, and TCGA databases, which are based on clinical outcomes and gene expression. As shown in Figure 3A and Supplementary Figures S2A–H, the expression level of *SYDE1* also increased with increasing WHO grade (*p* < 0.05). The protein expression pattern of *SYDE1* in gliomas described by the Human Protein Atlas also helped account for this characteristic. As shown in Figures 4A–C, SYDE1 protein expression was moderate in LGG, and strong expression was detected in HGG tissues. This result is consistent with the mRNA microarrays. In the GSE4290 and GSE4412_GPL96 datasets, the expression level of *SYDE1* increased in the order of control of oligodendrogliomas, astrocytomas, and GBMs. The expression of *SYDE1* in GBMs was also highest in the other histologies in the GSE68848 dataset (*p* < 0.05, Figure 3B, Supplementary Figures S2I, SJ). Furthermore, we also performed IHC to investigate SYDE1 expression in glioma tissue. Intriguingly, our results also exhibited such characteristics (Table 2). As shown in Figures 5A–F, positive staining of SYDE1 was predominantly found in grade IV glioma compared with grade I glioma. Therefore, SYDE1 strongly correlated with WHO glioma grade at the mRNA and protein levels.

**TABLE 2:** Association between *SYDE1* expression and WHO grade in glioma.

Group	WHO grade					Fisher’s exact test	*p*-value
	Normal	I	II	III	IV		
Low expression of *SYDE1*	4	4	7	6	3	10.615	0.021^*^
High expression of *SYDE1*	1	1	3	4	12		

### *SYDE1* Expression Significantly Differentiated Glioma Subtypes and Was Statistically Associated With Molecular Genetic Features

**FIGURE 6:**
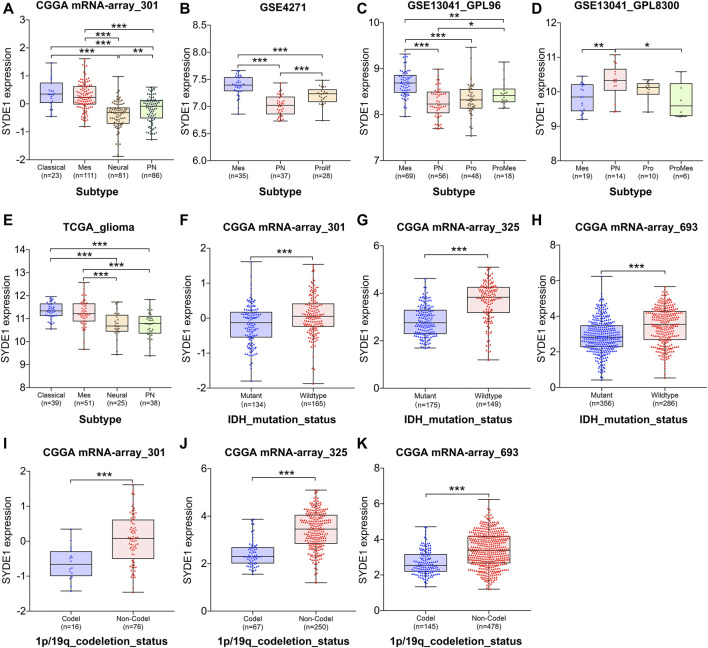
The association between *SYDE1* expression and glioma subtypes, IDH mutation or 1p/19q co-deletion. **(A–E)** The association between *SYDE1* expression and glioma subtypes. **(F–H)** The association between *SYDE1* expression and IDH mutation. **(I–K)** The association between *SYDE1* expression and 1p/19q co-deletion. *, p.value <0.05; **, p.value <0.01; ***, p.value <0.001.

Initially, *SYDE1* correlated with clinical parameters, and the expression levels of *SYDE1* were significantly associated with glioma WHO grade. Therefore, it is tempting to speculate that *SYDE1* expression correlates with glioma subtype. Several molecular biomarkers, including the IDH mutation and chromosome 1p/19q codeletion, are used for classification of gliomas. These biomarkers support the basis for individual glioma clinical therapy and molecular targeted therapy. Based on these markers, different molecular classifications were proposed. Therefore, we further analyzed the relationship between *SYDE1* expression and different classification subtypes. Our results showed that *SYDE1* was more highly expressed in Mes subtypes than neural, PN, and Prolif subtypes based on CGGA mRNA-array_301, GSE4271, GSE13041_GPL96, and TCGA_glioma datasets (*p* < 0.05, Figures 6A–C,E), but this association was not pronounced in GSE13041_GPL8300 (Figure 6D). Another part of the data (CGGA mRNA-array_301, GSE4271, GSE13041_GPL96, and TCGA_glioma dataset) suggested that *SYDE1* expression was significantly lower in the neural or PN subtypes (*p* < 0.05, Figures 6A–C,E). However, the ProMes subtypes had the lowest *SYDE1* expression of all subtypes in the GSE13041_GPL8300 dataset (*p* < 0.05, Figure 6D). We also noted that *SYDE1* was differentially expressed in various subtypes between the CGGA, TCGA, and GEO databases (*p* < 0.05, Figures 6A–E).

Notably, *SYDE1* expression strongly correlated with favorable prognostic factors (i.e., IDH mutation and chromosome 1p/19q codeletion). The analyzed data (CGGA mRNA_301, CGGA mRNA_325, and CGGA mRNA_693) indicated that glioma patients with the IDH_mutation attribute exhibited lower *SYDE1* expression than patients with IDH_wildtype (*p* < 0.001, Figures 6F–H). Similarly, the 1p/19q codeletion demonstrated the same correlation, which means that *SYDE1* expression was decreased in the 1p/19q_codeletion glioma samples compared with the corresponding 1p/19q_noncodeletion samples (*p* < 0.05, Figures 6I–K).

### The Unfavorable Prognostic Role of *SYDE1* Expression in Glioma Patients

**FIGURE 7:**
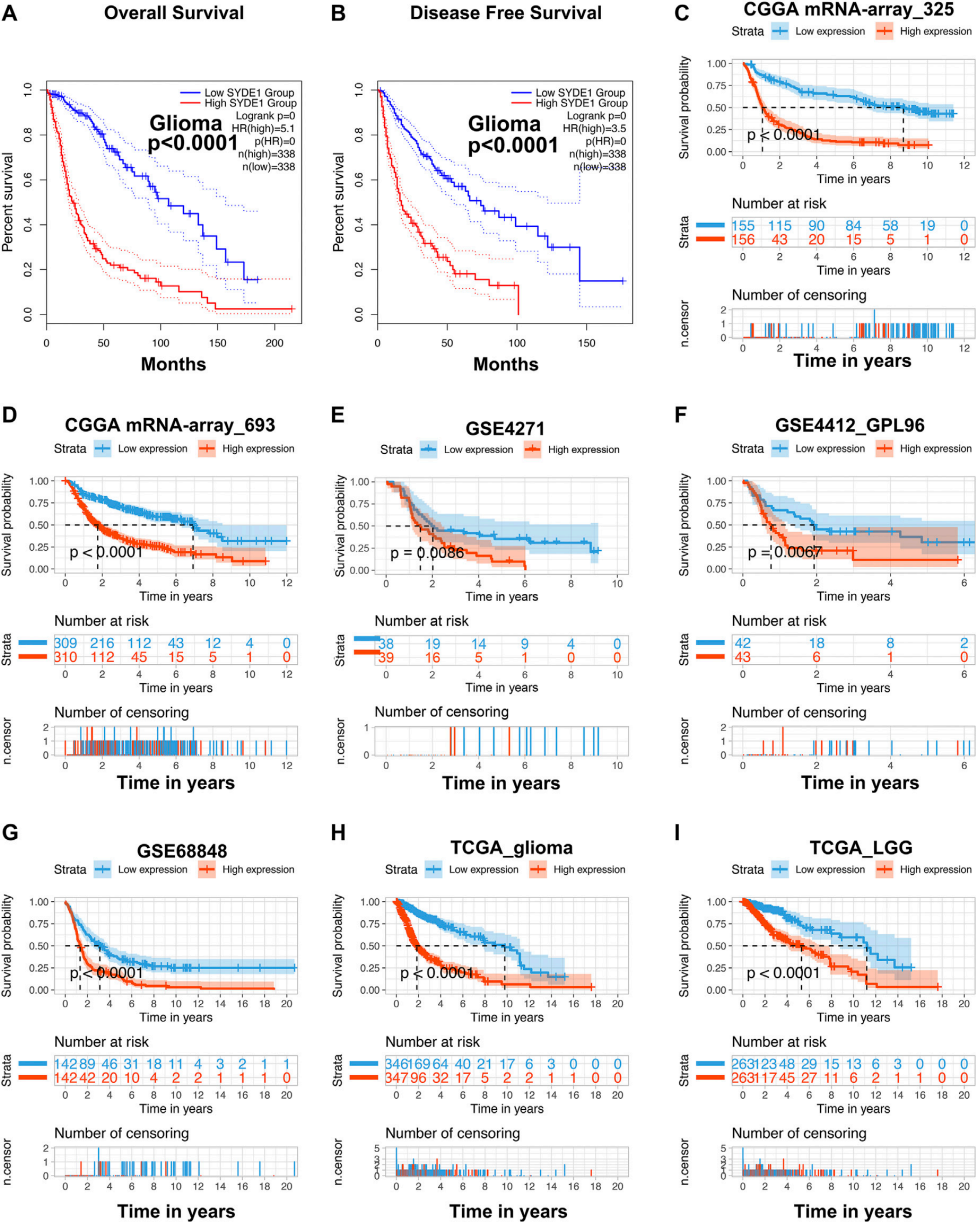
Evaluation of the prognostic value of *SYDE1* in different datasets. **(A–B)** Patients with low *SYDE1* expression groups had a long median OS and DFS than high *SYDE1* expression groups, respectively (GEPIA2). **(C–I)** The high expression of *SYDE1* was positively related to inferior prognosis regarding OS by data mining in different datasets, including mRNA-array_325, mRNA-array_693 of CGGA, GSE4271, GSE4412_GPL96, GSE68848, TCGA_glioma, and TCGA_LGG, respectively.

We investigated the prognostic ability of *SYDE1* for overall survival (OS) and disease-free survival (DFS) using the GEPIA2 online database. As expected, individuals with increased *SYDE1* in gliomas were associated with poor OS and DFS (*p* < 0.0001, Figures 7A,B). The TCGA, CGGA, and GEO cohorts also supported the result that high *SYDE1* expression in gliomas is associated with shorter OS time, and decreased levels of *SYDE1* expression are associated with longer OS time in glioma patients (*p* < 0.05, Figures 7C–I).

### Cox Regression Analysis Revealed That *SYDE1* May Be an Independent Survival Factor in Gliomas

After validation of the prognostic function of *SYDE1* in gliomas, we further analyzed the existing data in the CGGA and TCGA databases to confirm whether it was an independent survival predictor in individuals with gliomas. The results of univariate Cox regression analysis demonstrated that some parameters, including *SYDE1* expression, age, WHO grade, primary/recurrent/secondary (PRS) type, histology, radiotherapy, chemotherapy, IDH mutation, and 1p/19q codeletion status, were independent risk factors for the prognosis prediction in glioma patients based on the CGGA mRNA-array_325. Multivariate Cox regression analysis demonstrated that *SYDE1* expression, age, WHO grade, PRS type, chemotherapy, and IDH mutation were independent risk factors for OS (*p* < 0.05, Supplementary Table S7). Notably, a similar result showed these features in the CGGA mRNA-array_693, TCGA_glioma, and TCGA_LGG datasets (*p* < 0.05, Supplementary Tables S8–S10). Based on these statistical results, *SYDE1* can be considered an independent prognostic risk factor in gliomas, at least in part.

### Knockdown of *SYDE1* Suppressed Migratory and Invasive, but Not Proliferative Abilities of Glioma Cells *in vitro*

**FIGURE 8:**
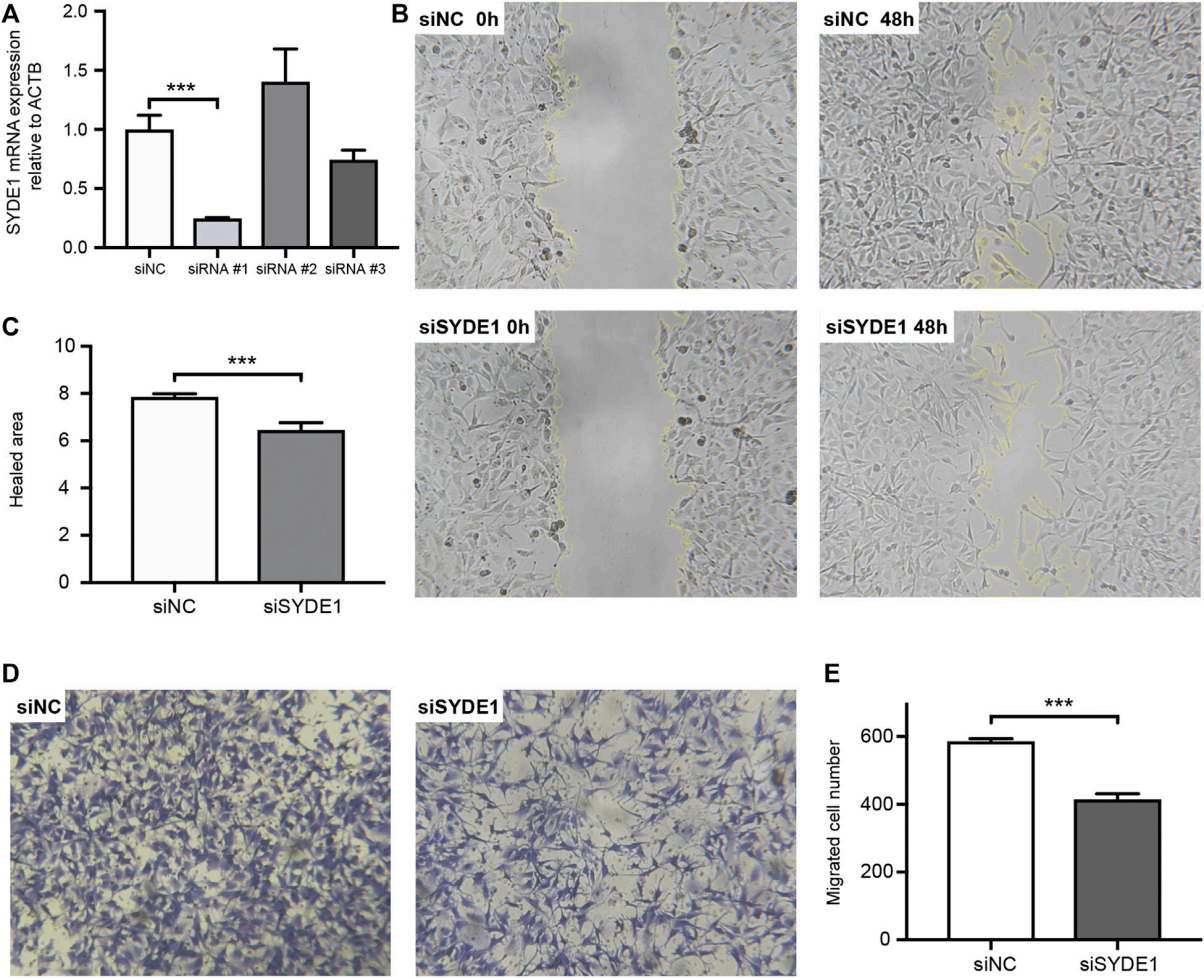
Wound scratch assay and transwell assay. **(A)** qPCR results indicate that knockdown of *SYDE1* with siSYDE1 #1 achieves the highest efficiency. Data are presented as mean ± SD, n = 3 per group, ***, p.value <0.001 for siSYDE1 #1 group versus control group. **(B)** Representative images of A172 migration in the siNC and siSYDE1 groups at 0 and 48 h in the scratch assay. **(C)** Bar graph illustrating the number of cells per high field. Data are presented as mean ± SD, n = 3 per group, ***, p.value <0.001 for siSYDE1 group versus control group. (D) Representative images of invasive cells in the lower chamber stained with crystal violet in the siNC and siSYDE1 groups in the transwell assay. **(E)** Bar graph illustrating the percentage of the scratched area covered. Data are presented as mean ± SD, n = 3 per group, ***, p.value <0.001 for siSYDE1 group versus control group.

To date, the role of *SYDE1* in glioma development remains largely undetermined. Based on the correlation between *SYDE1* expression and glioma revealed by bioinformatic analysis and IHC, we performed *in vitro* experiments to further verify this association. To achieve this, we first tested three different siRNA oligonucleotide sequences for their efficiency in suppressing *SYDE1* in A172 cells. After 24 h of siRNA transfection into human astroglial A172 cells, analysis of *SYDE1* expression by qPCR indicated that siRNA#1 was the most effective, mediating 75.6 ± 0.9% mRNA knockdown (*p* < 0.01) compared with nontargeting siRNA (Figure 8A). The knockdown of *SYDE1* failed to affect *SYDE1* cell proliferation in the CCK-8 viability assay (Supplementary Figure S3). Then, wound healing and transwell assays were performed, which revealed that *SYDE1* knockdown significantly reduced the healed area at 24 h (Figures 8B,C) (*p* < 0.01) and the number of migrated cells at 24 h (Figures 8D,E) (*p* < 0.01).

### Prediction of Coexpression Genes for *SYDE1* Using cBioPortal

**FIGURE 9:**
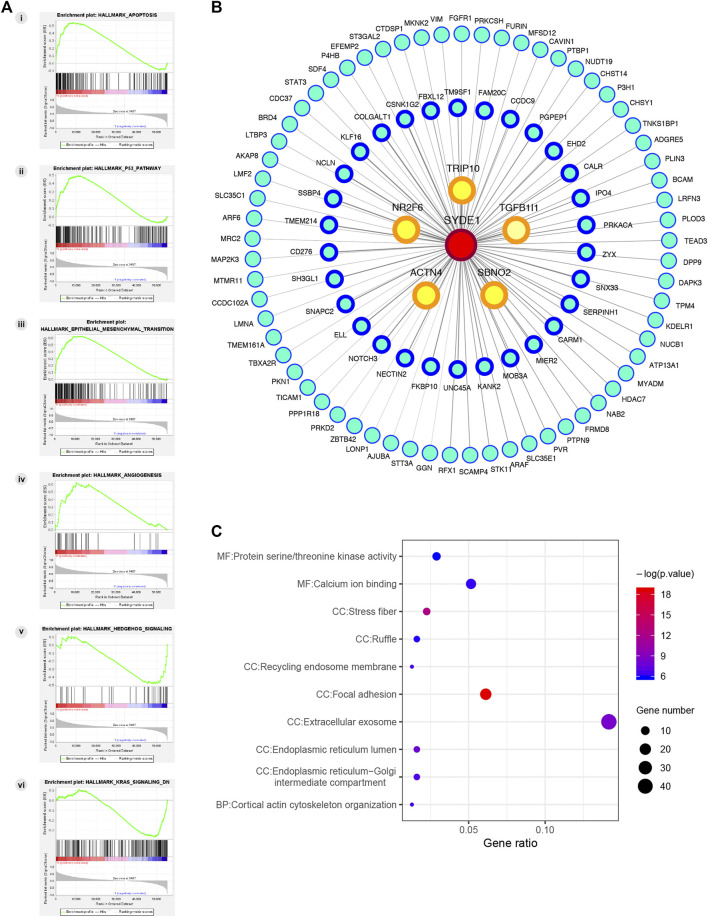
Potential molecular mechanism of *SYDE1* in glioma. **(A)** GSEA pathway using single-gene method of *SYDE1*. (i-iv) Enriched gene sets in the group of high *SYDE1* expression. (v-vi) Enriched gene sets in the group of low *SYDE1* expression. **(B)** The PPI network of the top 100 genes co-expressed with *SYDE1* in glioma tissues. **(C)** GO enrichment results of 100 genes involved in PPI network.

GSEA has served as an effective strategy for determining the biological function of a novel gene implicated in tumor development (Wang et al., 2019). To preliminarily decipher the biological function of *SYDE1* in glioma, GSEA was performed to identify gene sets enriched in the high- and low *SYDE1* expression groups. In the high *SYDE1* expression group, significantly enriched hallmark gene sets were associated with tumorigenesis and metastasis, including “Epithelial Mesenchymal Transition”, “p53 pathway”, “Apoptosis” and “Angiogenesis” (Figure 9A i-9A iv). In the low *SYDE1* expression group, tumor-associated terms “Hedgehog signaling” and “Kras signaling” were the enriched gene sets (Figure 9A v-9A vi). As shown in Figures 9A,B comprehensive coexpression network was built according to the top 100 Spearman’s correlation index values from the cBioPortal databases. The top-ranking genes were related to the TRIP10, NR2F6, ACTN4, SBNO2, and TGFB1L1 genes (*p* < 0.05), and a link between ACTN4 and TGFB1L1 expression and tumorigenesis was shown in glioma samples (Ji et al., 2019). GO enrichment analysis was conducted for the coexpressed genes of *SYDE1* (*p* < 0.05, Figure 9C), and the coexpressed genes primarily participated in the molecular functions of protein serine kinase activity and calcium ion binding. The coexpressed genes were involved in various cellular components, including stress fiber, ruffle, recycling endosome membrane, focal adhesion, extracellular exosome, and endoplasmic reticulum lumen. Enriched signaling pathways for the top 100 coexpressed genes of *SYDE1* identified in the KEGG pathway analysis were ranked according to *p* values. As shown in Table 3, the insulin signaling pathway, adherens junctions, and protein processing in the endoplasmic reticulum remained particularly significant. Reactome pathway analysis was further used to identify the metabolic pathways in which the top 100 coexpressed genes were related to *SYDE1* with *p* < 0.05. The top 10 critically enriched Reactome pathways were obtained and are presented in Table 4.

**TABLE 3:** Kyoto Encyclopedia of Genes and Genomes (KEGG) pathway analysis for predicted co-expression genes of *SYDE1* in gliomas.

Term	ID	Count	*p*-Value	FDR	Fold enrichment	Genes
Insulin signaling pathway	ptr04910	4	0.041691	38.844556	5.033333333	ARAF, MKNK2, PRKACA, TRIP10
Adherens junction	ptr04520	3	0.062905	52.77482	7.13630137	FGFR1, ACTN4, NECTIN2
Protein processing in endoplasmic reticulum	ptr04141	4	0.066436	54.789246	4.159281437	P4HB, STT3A, CALR, PRKCSH
Dilated cardiomyopathy	ptr05414	3	0.086996	65.040685	5.919886364	LMNA, PRKACA, TPM4

**TABLE 4:** Reactome pathways analysis for predicted co-expression genes of *SYDE1* in gliomas.

Pathway identifier	Pathway name	Entities *p*-Value	Entities FDR	Submitted entities found
R-HSA-1474244	Extracellular matrixorganization	1.97E–05	0.010111351	EFEMP2;SERPINH1;P3H1;PLOD3;FURIN;LTBP3;P4HB;PRKACA;COLGALT1
R-HSA-1650814	Collagen biosynthesis andmodifying enzymes	2.50E–05	0.010111351	SERPINH1;P3H1;PLOD3;P4HB;COLGALT1
R-HSA-1474290	Collagen formation	1.74E–04	0.035258571	SERPINH1;P3H1;PLOD3;P4HB;COLGALT1
R-HSA-1566948	Elastic fibre formation	1.75E–04	0.035258571	EFEMP2;FURIN;LTBP3
R-HSA-5655302	Signaling by FGFR1in disease	5.00E–04	0.080577744	PTBP1;STAT3;FGFR1
R-HSA-2129379	Molecules associatedwith elastic fibres	8.93E–04	0.119642788	EFEMP2;LTBP3
R-HSA-1839124	FGFR1 mutant receptoractivation	0.002088233	0.223660696	STAT3;FGFR1
R-HSA-4419969	Depolymerisation of theNuclear Lamina	0.002214462	0.223660696	LMNA;PRKACA
R-HSA-420597	Nectin/Necl transheterodimerization	0.002812629	0.232344096	PVR;NECTIN2
R-HSA-5663202	Diseases of signal transductionby growth factor receptorsand second messengers	0.002904301	0.232344096	PTBP1;NOTCH3;ATP13A1;CDC37;STAT3;LMNA;ARAF;FGFR1;HDAC7

### Construction of the mRNA/miRNA/lncRNA Network

**FIGURE 10:**
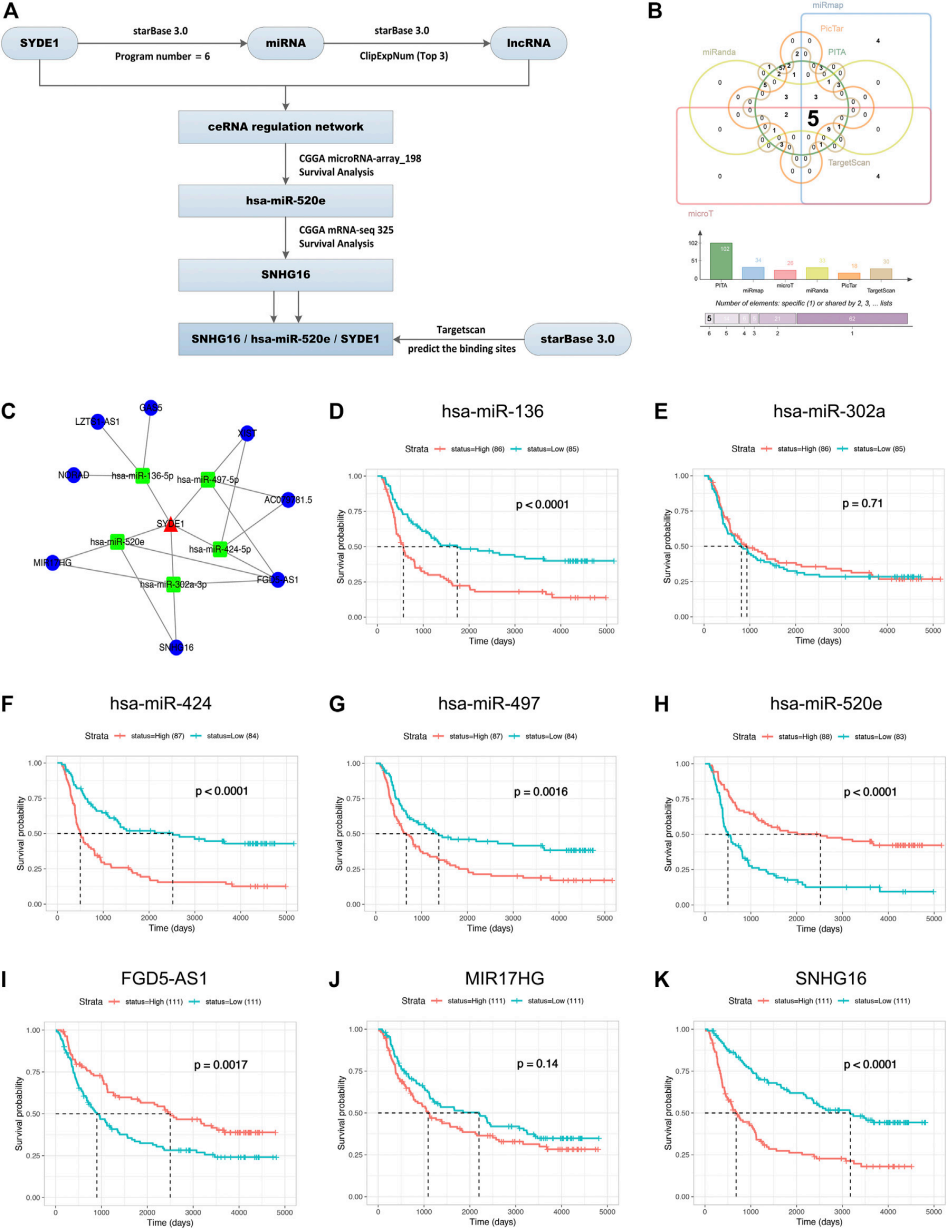
Prediction of *SYDE1*-targeted miRNAs and lncRNAs. **(A)** A schematic overview of different steps taken to predict *SYDE1*-targeted miRNA and lncRNA. **(B)** Upset plot showing the number of predicted *SYDE1*-targeted miRNAs. **(C)** Network of *SYDE1-*targeted miRNAs and lncRNAs. **(D–H)** Kaplan-Meier survival curves for 5-miRNA signature. has-miR-136 **(D)**, has-miR-424 **(F)**, has-miR-497 **(G)** and has-miR-520e **(H)** are positively associated with OS, whereas has-miR-302a **(E)** is not associated with OS. (I–K) Kaplan-Meier survival curves for 3-lncRNA signature. FGD5-AS1 **(I)**, MIR17HG **(J)** and SNHG16 **(K)** are positively associated with OS.

We used a comprehensive strategy for starBase 3.0, CGGA, and TargetScan databases to investigate the potential miRNA targets and the indirect lncRNAs targets of *SYDE1* in gliomas. Recapitulation of this part of the work is shown in Figure 10A as a flow chart. A total of 143 target miRNAs were acquired from the PITA, miRmap, microT, miRanda, PicTar, and TargetScan databases. By intersecting the predicted target miRNAs, we created a Venn diagram to identify five individual miRNAs that were predicted by all six databases from the 143 miRNA assemblies. There were 14 target miRNAs of *SYDE1* that were supported by at least five databases, and six miRNAs were predicted by the four most common databases (Figure 10B). The lncRNAs predicted by target miRNAs in starBase 3.0 were identified for the construction and integration of the mRNA-miRNA-lncRNA network, which may offer intuitive insight into the competing endogenous RNA (ceRNA) mechanism in gliomas (Figure 10C). The five optimal target miRNAs screened by the six databases were tested for relevance between the expression level and survival. Taking into consideration the clinical features from the CGGA dataset (miRNA-array_198 and CGGA mRNA-seq 325), the survival curves showed that patients with upregulation of hsa-miR-520e had a favorable OS in primary gliomas (*p* < 0.05, Figure 10H), but this generalization was not true for the other four miRNAs (hsa-miR-136, hsa-miR-302a, hsa-miR-424, and hsa-miR-497, Figures 10D–G). Considering that hsa-miR-520e is the target miRNA of *SYDE1* with favorable outcomes in gliomas, the expression of the target lncRNAs (FGD5-AS1, MIR17HG, and SNHG16) of hsa-miR-520e was measured to calculate the prognostic ability of these signatures. Expression of SNHG16 was higher in LGGs or HGGs than normal brain tissues (Supplementary Figure S4). The results demonstrated that the high expression of SNHG16 in primary glioma patients had a lower OS (*p* < 0.05, Figure 10K), and in the other two lncRNA (FGD5-AS1, MIR17HG) groups, there was no apparent defined association described for SNHG16 (Figures 10I,J). These results provided initial evidence for the *SYDE1/hsa-miR-520e/SNHG16* network in gliomas.

### Identification of *SYDE1/Hsa-miR-520e/SNHG16* Interactions

Hsa-miR-520e and SNHG16 were selected for further evaluation. A growing body of evidence demonstrates that lncRNAs play a key role in tumorigenesis by functioning as competing endogenous RNAs (ceRNAs) to mRNA. To delineate the precise *SYDE1*/hsa-miR-520e/SNHG16 interaction, the potential targets were collected from the PITA, miRmap, microT, miRanda, PicTar, TargetScan, and starBase 3.0 databases. Six databases identified *SYDE1*/hsa-miR-520e interactions, and one database identified an hsa-miR-520e/SNHG16 interaction (Supplementary Figure S5).

## Discussion

Gliomas are the most common primary malignant brain tumors in adults, and their exact molecular mechanisms have not yet been completely elucidated. In this study, we report the gene *SYDE1* as a novel regulator of glioma tumors. First, we analyzed publicly published mRNA expression data of human glioma tissues and normal control tissues and found that *SYDE1* expression was higher in gliomas than in healthy cerebral tissues. Then, normal human brain and glioma samples from grades I to IV were collected for IHC staining of SYDE1, which revealed that SYDE1 expression is positively correlated with the clinical malignancies of glioma. In addition, *SYDE1* is more highly expressed in recurrent or necrotic gliomas or gliomas that occur in elderly patients.

A difference in the expression of a gene between normal brain tissue and glioma tissues indicates a potential modulatory mechanism of glioma development. There are many well-studied pathogenic genes and regulators of gliomas, including IDH1/2, 1p/19q codeletion, integrin β1 (ITGB1) and v-raf murine sarcoma viral oncogene homolog B1 (BRAF) V600E mutation (Chintala et al., 1996; Schiffman et al., 2010; Yip et al., 2012). In particular, ITGB1 encodes the β1 subunit of extracellular matrix (ECM) integrins and shows reduced expression in gliomas compared with normal controls. Treating a human glioblastoma cell line with anti-β1 antibodies *in vitro* diminishes integrin synthesis on the cell surface, which can result in an increase in matrix metalloprotease-2 activity and invasiveness of the cell (Chintala et al., 1996). In a similar manner, our data show that the expression of *SYDE1* is higher in human glioma tissues, so it is reasonable to explore the biological function and molecular mechanism of *SYDE1* in gliomas.

With regard to the molecular mechanism, it is noteworthy that GSEA of the high *SYDE1* expression group showed enrichments in epithelial mesenchymal transition and the p53 pathway, which are highly related to tumorigenesis and metastasis. Moreover, GO and KEGG pathway analysis of *SYDE1* coexpressed genes revealed an enrichment of tumor-associated terms, including protein serine kinase activity and focal adhesion. Of note, adhesion to and migration through the extracellular matrix (ECM) is recognized as an important part of the metastatic process and is necessary for the invasion of a variety of tumors (Pauli et al., 1983). The role of SYDE1 in modulating cell migration has already been reported in recent years. As revealed by Lo et al., SYDE1 can promote cytoskeletal remodeling as well as migration and invasion of placental trophoblast cells, which is crucial for maintaining the maternal-trophoblast interface (Lo et al., 2017). In our study, knockdown of *SYDE1 in vitro* significantly abolished the migration and invasion of glioma cell lines A172. Taken together, increased expression of *SYDE1* in gliomas may lead to an overactivated transcriptional network that facilitates tumor invasion.

We next report the SNHG16/hsa-miR-520e axis as a downstream target of *SYDE1* in gliomas. SNHG16 is a novel cancer-related lncRNA and has been demonstrated to function as an oncogene in human breast cancer, gastric cancer, or hepatocellular cancer (Yang and Wei, 2019). For instance, increased SNHG16 expression in human gastric cancer can promote *in vitro* proliferation and *in vivo* growth of gastric tumors (Lian et al., 2017). SNHG16 is also reported to be downregulated in several other malignant tumors, such as hepatocellular carcinoma, and to inhibit tumor growth (Lin et al., 2019). Considering the versatile role of SNHG16 in tumor development, we examined SNHG16 expression in our study and found that the SNHG16 level was significantly increased in gliomas. That is, upregulated *SYDE1* in gliomas can potentially activate SNHG16 expression to facilitate the onset and progression of gliomas. microRNAs (miRNAs), such as hsa-miR-93, miR-338-3p, miR-124-3p and miR-128, are common targets of SNHG16 in human cancers (Xu et al., 2018; Chen et al., 2020; Wu et al., 2020). In our study, hsa-miR-520e was identified to interact with SNHG16 in gliomas via RMBase 2.0, and further *in vitro* experiments to validate the SNHG16/hsa-miR-520e axis are in progress.

In summary, this study identifies *SYDE1* as an oncogene in gliomas that can regulate the proliferation and migration of glioma cells and is predicted to interact with the SNHG16/hsa-miR-520e axis. In-depth elucidation of the functions of *SYDE1* in gliomas, such as tumor formation in nude mice and population mutation screening, will provide a deeper understanding of the molecular pathology of gliomas. With these advances, *SYDE1* can be a promising future biomarker for glioma clinical practices, including serving as a reference for the WHO classification and predicting the prognosis of glioma patients.

## Data Availability

The datasets presented in this study can be found in online repositories. The names of the repository/repositories and accession number(s) can be found in the article/Supplementary Material.
