# Supplementary material for: SYDE1 Acts as an Oncogene in Glioma and has Diagnostic and Prognostic Values
Source: Front Mol Biosci. 2021 Oct 14;8:714203. doi: 10.3389/fmolb.2021.714203 (PMC8552071; doi:10.3389/fmolb.2021.714203)
Supplement: Supplementary file 1 [file DataSheet1.docx]

Supplementary Material

**Supplemental Figure S1** The association between *SYDE1* expression and patient age, or tumor recurrence. (A-C) The association between *SYDE1* expression and patient age. (D) The association between *SYDE1* expression and glioma recurrence. *, p.value < 0.05; ***, p.value < 0.001.**
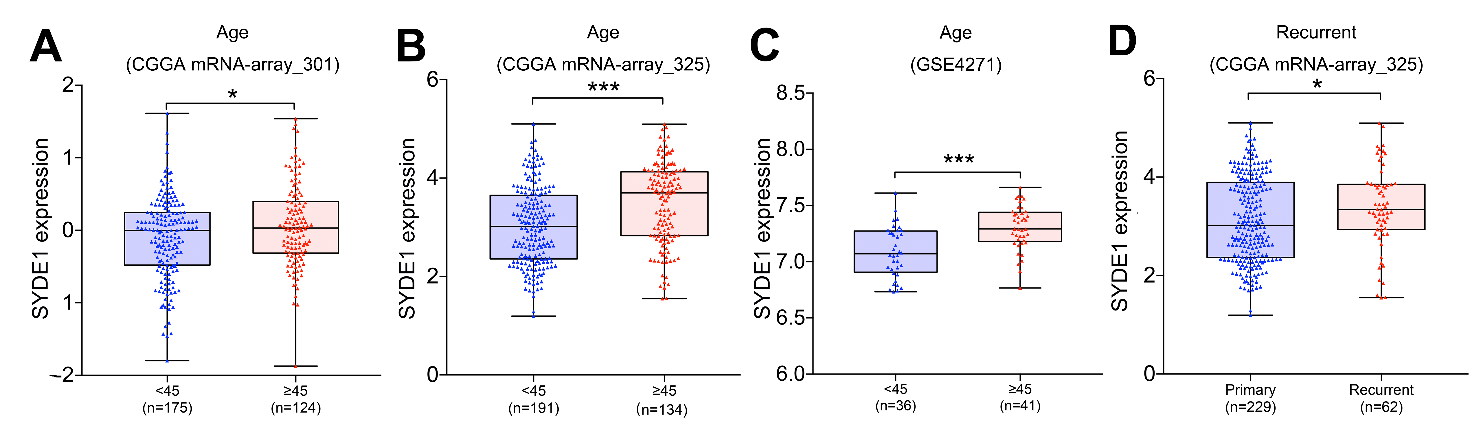
**

**Supplemental Figure S2** The association between *SYDE1* expression and glioma grade or histology. (A-H) The association between *SYDE1* expression and glioma grades. (I-J) The association between *SYDE1* expression and glioma histology. *, p.value < 0.05; **, p.value < 0.01; ***, p.value < 0.001.
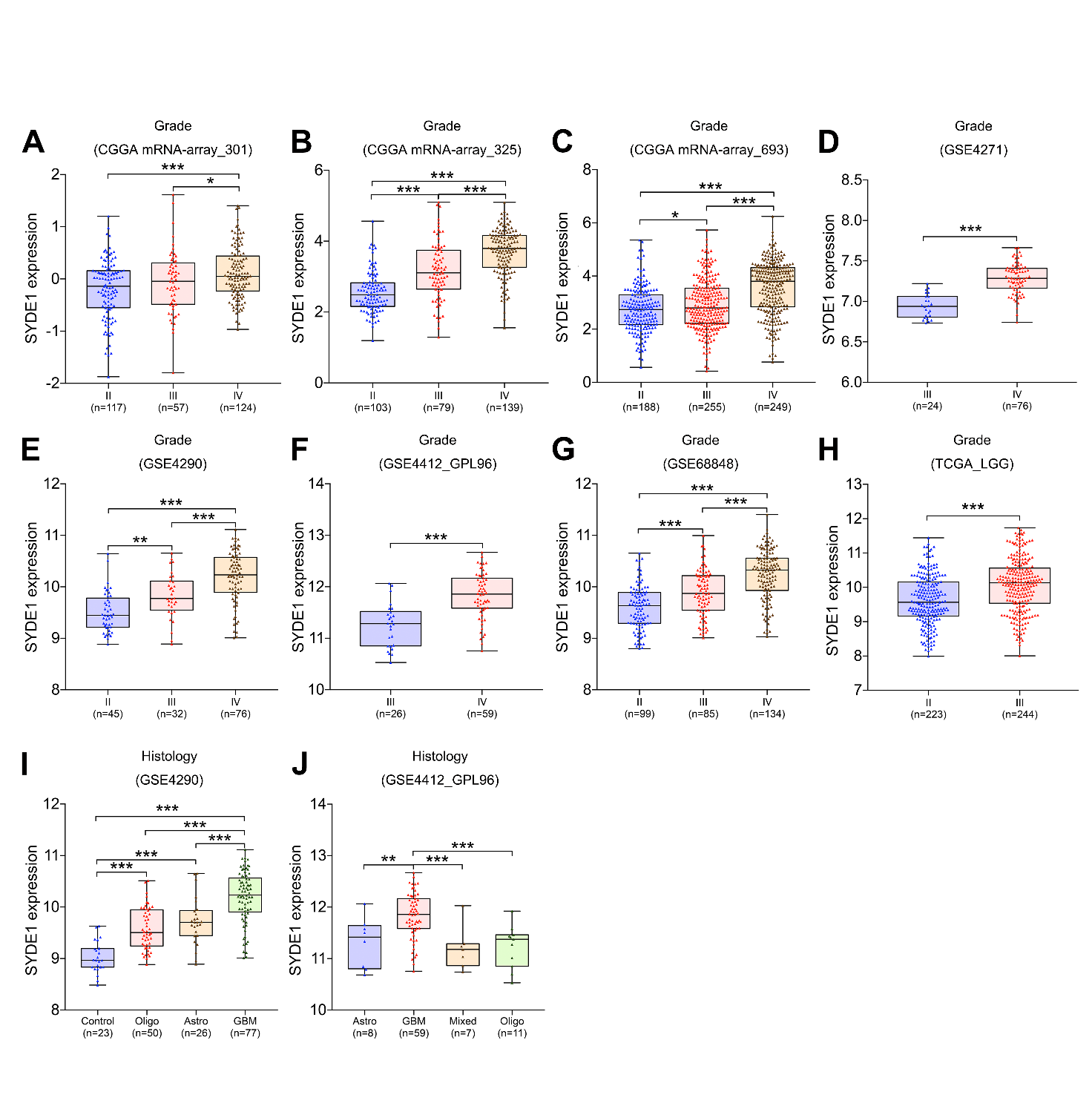


**Supplemental Figure 3** Line graph illustrating cell density in the control and siSYDE1 groups across time in the CCK8 assay. NS, p.value> 0.05.


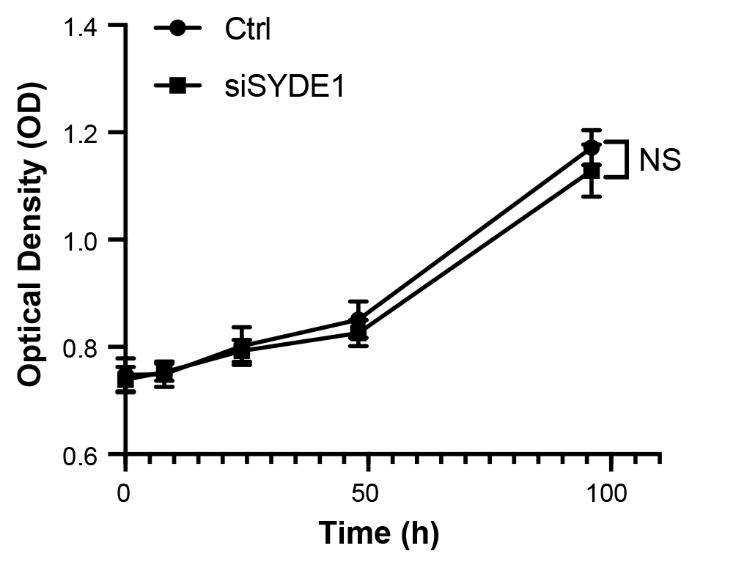


**Supplemental Figure 4** SNHG16 expression in normal human brains, LGGs and GBMs. *, p.value < 0.05; **, p.value < 0.01; ***, p.value < 0.001.


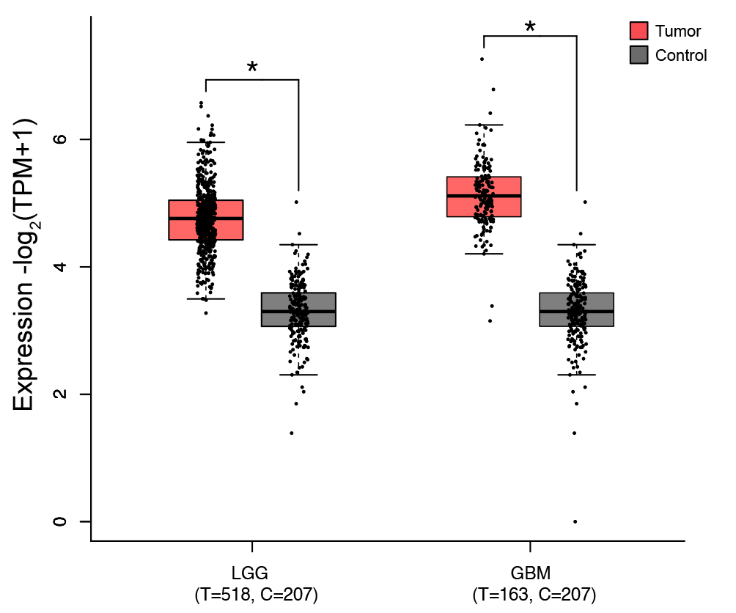


**Supplemental Figure 5** hsa-miR-520e is predicted to be the target of *SYDE1* (A-F). SNHG16 is predicted to be the target of hsa-miR-520e (G).


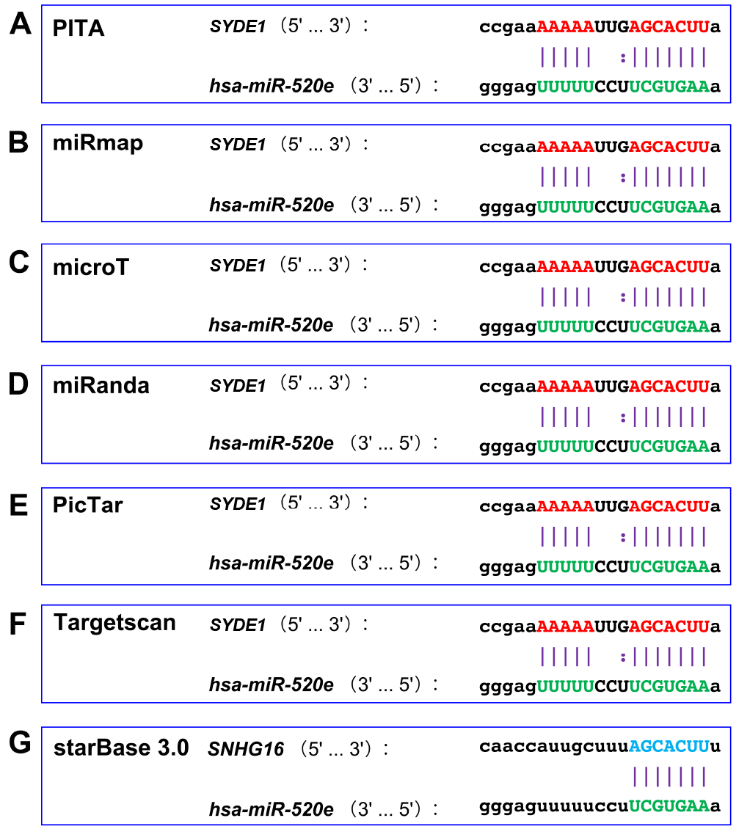


**Supplemental Table 1** Correlation of *SYDE1* with clinicopathologic characteristics of glioma patients in CGGA mRNA-array_301.

| Variables | Case  (n=301) | SYDE1 | | P value |
| --- | --- | --- | --- | --- |
|  |  | Low (n=140) | High (n=161) |  |
| Age (years) |  |  |  | 0.391 |
| <45 | 175 | 84 | 90 |  |
| ≥45 | 124 | 54 | 70 |  |
| NA | 2 | 1 | 1 |  |
| Gender |  |  |  | 0.380 |
| Female | 121 | 60 | 62 |  |
| Male | 180 | 80 | 100 |  |
| WHO grade |  |  |  | 0.159 |
| Ⅱ | 187 | 61 | 56 |  |
| Ⅲ | 57 | 29 | 28 |  |
| Ⅳ | 124 | 50 | 74 |  |
| NA | 3 | 0 | 3 |  |
| Histology |  |  |  | **P<0.001** |
| A | 58 | 11 | 47 |  |
| AA | 12 | 5 | 7 |  |
| AO | 10 | 6 | 4 |  |
| AOA | 22 | 11 | 11 |  |
| GBM | 108 | 43 | 65 |  |
| O | 18 | 13 | 3 |  |
| OA | 36 | 34 | 2 |  |
| rA | 5 | 1 | 4 |  |
| rAA | 4 | 2 | 2 |  |
| rAO | 4 | 3 | 1 |  |
| rAOA | 5 | 2 | 3 |  |
| rGBM | 5 | 3 | 2 |  |
| sGBM | 11 | 3 | 7 |  |
| NA | 3 | 0 | 3 |  |
| PRS_type |  |  |  | 0.772 |
| Primary | 264 | 115 | 139 |  |
| Recurrent | 23 | 11 | 12 |  |
| Secondary | 11 | 4 | 7 |  |
| NA | 3 | 0 | 3 |  |
| TCGA_subtypes |  |  |  | **P<0.001** |
| Classical | 23 | 5 | 18 |  |
| Mesenchymal | 111 | 26 | 85 |  |
| Neural | 81 | 62 | 19 |  |
| Proneural | 86 | 37 | 39 |  |
| Radio_status |  |  |  | 0.073 |
| Negative | 38 | 13 | 25 |  |
| Positive | 249 | 124 | 125 |  |
| NA | 14 | 3 | 11 |  |
| Chemo_status |  |  |  | 0.223 |
| Negative | 126 | 66 | 60 |  |
| Positive | 151 | 68 | 83 |  |
| NA | 24 | 6 | 18 |  |
| IDH_mutation_status |  |  |  | 0.091 |
| Mutant | 134 | 70 | 64 |  |
| Wildtype | 165 | 70 | 95 |  |
| NA | 2 | 0 | 2 |  |
| 1p19q_Codeletion |  |  |  | **0.001** |
| Codel | 16 | 14 | 2 |  |
| Non-codel | 76 | 33 | 43 |  |
| NA | 209 | 93 | 116 |  |

Note: CGGA, the Chinese Glioma Genome Altas; WHO, World Health Organization; A, astrocytomas; AA, anaplastic astrocytomas; AO, anaplastic oligodendrogliomas; AOA, anaplastic oligoastrocytomas; GBM, glioblastoma multiforme; O, oligodendrogliomas; OA, oligoastrocytomas; rA, recurrent astrocytomas; rAA, recurrent anaplastic astrocytomas; rAO, recurrent anaplastic oligodendrogliomas; rAOA, recurrent anaplastic oligoastrocytomas; rGBM, recurrent glioblastoma multiforme; sGBM, secondary glioblastoma multiforme; TCGA, The Cancer Genome Atlas; NA, not analyze.

**Supplemental Table 2** Correlation of *SYDE1* with clinicopathologic characteristics of glioma patients in CGGA mRNA-array_325.

| Variables | Case  (n=325) | *SYDE1* | | P value |
| --- | --- | --- | --- | --- |
|  |  | Low (n=163) | High (n=162) |  |
| Age (years) |  |  |  | **P<0.001** |
| <45 | 191 | 112 | 79 |  |
| ≥45 | 134 | 51 | 83 |  |
| Gender |  |  |  | 0.183 |
| Female | 122 | 67 | 55 |  |
| Male | 203 | 96 | 107 |  |
| WHO grade |  |  |  | **P<0.001** |
| Ⅱ | 103 | 89 | 14 |  |
| Ⅲ | 79 | 43 | 36 |  |
| Ⅳ | 139 | 31 | 108 |  |
| NA | 4 | 0 | 4 |  |
| Histology |  |  |  | **P<0.001** |
| A | 33 | 30 | 3 |  |
| AA | 14 | 7 | 7 |  |
| AO | 9 | 8 | 1 |  |
| AOA | 27 | 13 | 14 |  |
| GBM | 85 | 14 | 71 |  |
| O | 26 | 25 | 1 |  |
| OA | 35 | 31 | 4 |  |
| rA | 6 | 1 | 5 |  |
| rAA | 14 | 6 | 8 |  |
| rAO | 3 | 2 | 1 |  |
| rAOA | 12 | 7 | 5 |  |
| rGBM | 24 | 6 | 18 |  |
| rOA | 3 | 2 | 1 |  |
| sGBM | 30 | 11 | 19 |  |
| NA | 4 | 0 | 4 |  |
| PRS_type |  |  |  | **0.015** |
| Primary | 229 | 128 | 101 |  |
| Recurrent | 62 | 24 | 38 |  |
| Secondary | 30 | 11 | 19 |  |
| NA | 4 | 0 | 4 |  |
| Radio_status |  |  |  | **0.007** |
| Negative | 51 | 17 | 34 |  |
| Positive | 258 | 139 | 119 |  |
| NA | 16 | 7 | 9 |  |
| Chemo_status |  |  |  | **0.002** |
| Negative | 124 | 79 | 51 |  |
| Positive | 178 | 77 | 101 |  |
| NA | 23 | 13 | 10 |  |
| IDH_mutation_status |  |  |  | **P<0.001** |
| Mutant | 175 | 124 | 51 |  |
| Wildtype | 149 | 38 | 111 |  |
| NA | 1 | 1 | 0 |  |
| 1p19q_Codeletion |  |  |  | **P<0.001** |
| Codel | 67 | 60 | 7 |  |
| Non-codel | 250 | 100 | 150 |  |
| NA | 8 | 3 | 5 |  |

Note: CGGA, the Chinese Glioma Genome Altas; WHO, World Health Organization; A, astrocytomas; AA, anaplastic astrocytomas; AO, anaplastic oligodendrogliomas; AOA, anaplastic oligoastrocytomas; GBM, glioblastoma multiforme; O, oligodendrogliomas; OA, oligoastrocytomas; rA, recurrent astrocytomas; rAA, recurrent anaplastic astrocytomas; rAO, recurrent anaplastic oligodendrogliomas; rAOA, recurrent anaplastic oligoastrocytomas; rGBM, recurrent glioblastoma multiforme; rOA, recurrent oligoastrocytomas; sGBM, secondary glioblastoma multiforme; NA, not analyze.

**Supplemental Table 3** Correlation of *SYDE1* with clinicopathologic characteristics of glioma patients in CGGA mRNA-array_693.

| Variables | Case  (n=693) | *SYDE1* | | P value |
| --- | --- | --- | --- | --- |
|  |  | Low (n=366) | High (n=327) |  |
| Age (years) |  |  |  | **0.047** |
| <45 | 382 | 215 | 167 |  |
| ≥45 | 312 | 153 | 159 |  |
| NA | 1 | 0 | 1 |  |
| Gender |  |  |  | 0.295 |
| Female | 295 | 149 | 146 |  |
| Male | 398 | 217 | 181 |  |
| WHO grade |  |  |  | **P<0.001** |
| Ⅱ | 188 | 134 | 54 |  |
| Ⅲ | 256 | 149 | 107 |  |
| Ⅳ | 249 | 83 | 166 |  |
| NA | 1 | 0 | 1 |  |
| Histology |  |  |  | **P<0.001** |
| A | 38 | 25 | 13 |  |
| AA | 34 | 19 | 15 |  |
| AO | 28 | 23 | 5 |  |
| AOA | 82 | 53 | 29 |  |
| GBM | 140 | 48 | 92 |  |
| O | 23 | 22 | 1 |  |
| OA | 78 | 59 | 19 |  |
| rA | 26 | 17 | 9 |  |
| rAA | 31 | 11 | 20 |  |
| rAO | 24 | 12 | 12 |  |
| rAOA | 58 | 33 | 25 |  |
| rGBM | 109 | 35 | 74 |  |
| rO | 7 | 4 | 3 |  |
| rOA | 17 | 7 | 10 |  |
| NA | 1 | 0 | 1 |  |
| PRS_type |  |  |  | **P<0.001** |
| Primary | 422 | 249 | 173 |  |
| Recurrent | 271 | 117 | 154 |  |
| Radio_status |  |  |  | **0.047** |
| Negative | 113 | 72 | 41 |  |
| Positive | 509 | 272 | 237 |  |
| NA | 71 | 22 | 49 |  |
| Chemo_status |  |  |  | **0.037** |
| Negative | 151 | 94 | 57 |  |
| Positive | 457 | 240 | 217 |  |
| NA | 85 | 32 | 53 |  |
| IDH_mutation_status |  |  |  | **P<0.001** |
| Mutant | 356 | 221 | 135 |  |
| Wildtype | 286 | 102 | 184 |  |
| NA | 51 | 43 | 8 |  |
| 1p19q_Codeletion |  |  |  | **P<0.001** |
| Codel | 145 | 107 | 38 |  |
| Non-codel | 478 | 197 | 281 |  |
| NA | 70 | 62 | 8 |  |

Note: CGGA, the Chinese Glioma Genome Altas; WHO, World Health Organization; A, astrocytomas; AA, anaplastic astrocytomas; AO, anaplastic oligodendrogliomas; AOA, anaplastic oligoastrocytomas; GBM, glioblastoma multiforme; O, oligodendrogliomas; OA, oligoastrocytomas; rA, recurrent astrocytomas; rAA, recurrent anaplastic astrocytomas; rAO, recurrent anaplastic oligodendrogliomas; rAOA, recurrent anaplastic oligoastrocytomas; rGBM, recurrent glioblastoma multiforme; rO, recurrent oligodendrogliomas; rOA, recurrent oligoastrocytomas; NA, not analyze.

**Supplemental Table 4** Correlation of *SYDE1* with clinicopathologic characteristics of glioma patients in GSE4271.

| Variables | Case  (n=100) | *SYDE1* | | P value |
| --- | --- | --- | --- | --- |
|  |  | Low (n=47) | High (n=53) |  |
| Age (years) |  |  |  | **0.005** |
| <45 | 36 | 22 | 14 |  |
| ≥45 | 41 | 12 | 29 |  |
| NA | 23 | 13 | 10 |  |
| Gender |  |  |  | 0.204 |
| Female | 32 | 18 | 14 |  |
| Male | 68 | 29 | 39 |  |
| WHO grade |  |  |  | **P<0.001** |
| Ⅲ | 24 | 23 | 1 |  |
| Ⅳ | 76 | 24 | 52 |  |
| Recurrence |  |  |  | 0.297 |
| Negative | 77 | 34 | 43 |  |
| Positive | 23 | 13 | 10 |  |
| Subtype |  |  |  | **P<0.001** |
| Mesenchymal | 35 | 5 | 30 |  |
| Proneural | 37 | 29 | 8 |  |
| Proliferative | 28 | 13 | 15 |  |
| Microvascular proliferation |  |  |  | **P<0.001** |
| Negative | 21 | 20 | 1 |  |
| Positive | 56 | 14 | 42 |  |
| NA | 23 | 13 | 10 |  |
| Necrosis |  |  |  | **P<0.001** |
| Negative | 27 | 22 | 5 |  |
| Positive | 50 | 12 | 38 |  |
| NA | 23 | 13 | 10 |  |

Note: WHO, World Health Organization; NA, not analyze.

**Supplemental Table 5** Correlation of *SYDE1* with clinicopathologic characteristics of LGG patients in TCGA.

| Variables | Case  (n=529) | *SYDE1* | | P value |
| --- | --- | --- | --- | --- |
|  |  | Low (n=261) | High (n=268) |  |
| Age (years) |  |  |  | 0.556 |
| <45 | 317 | 160 | 157 |  |
| ≥45 | 209 | 100 | 109 |  |
| NA | 3 | 1 | 2 |  |
| Gender |  |  |  | 0.949 |
| Female | 239 | 118 | 121 |  |
| Male | 288 | 143 | 145 |  |
| NA | 2 | 0 | 2 |  |
| Race |  |  |  | 0.226 |
| Asian | 8 | 4 | 4 |  |
| Black or african american | 22 | 7 | 15 |  |
| White | 486 | 246 | 240 |  |
| NA | 13 | 4 | 9 |  |
| WHO grade |  |  |  | **P<0.001** |
| Ⅱ | 223 | 144 | 79 |  |
| Ⅲ | 244 | 89 | 155 |  |
| NA | 62 | 28 | 34 |  |
| Histology |  |  |  | **P<0.001** |
| Astrocytoma, anaplastic | 129 | 26 | 103 |  |
| Astrocytoma, NOS | 66 | 28 | 38 |  |
| Mixed glioma | 132 | 70 | 62 |  |
| Oligodendroglioma, anaplastic | 82 | 49 | 33 |  |
| Oligodendroglioma, NOS | 118 | 88 | 30 |  |
| NA | 2 | 0 | 2 |  |
| KPS |  |  |  | 0.803 |
| <80 | 43 | 19 | 24 |  |
| ≥80 | 240 | 111 | 129 |  |
| NA | 246 | 131 | 115 |  |
| Treatment_or_therapy |  |  |  | **P<0.001** |
| Negative | 184 | 124 | 60 |  |
| Positive | 310 | 121 | 189 |  |
| NA | 35 | 16 | 19 |  |

Note: LGG, low-grade glioma; TCGA, The Cancer Genome Atlas; WHO, World Health Organization; KPS, Karnofsky performance score; NA, not analyze.

**Supplemental Table 6** Correlation of *SYDE1* with clinicopathologic characteristics of glioma patients in TCGA.

| Variables | Case  (n=698) | *SYDE1* | | P value |
| --- | --- | --- | --- | --- |
|  |  | Low (n=350) | High (n=348) |  |
| Age (years) |  |  |  | **P<0.001** |
| <45 | 337 | 213 | 124 |  |
| ≥45 | 357 | 135 | 222 |  |
| NA | 4 | 2 | 2 |  |
| Gender |  |  |  | 0.956 |
| Female | 298 | 150 | 148 |  |
| Male | 397 | 199 | 198 |  |
| NA | 3 | 1 | 2 |  |
| Race |  |  |  | 0.414 |
| Asian | 13 | 6 | 7 |  |
| Black or african american | 33 | 13 | 20 |  |
| White | 636 | 324 | 312 |  |
| NA | 16 | 7 | 9 |  |
| WHO grade |  |  |  | **P<0.001** |
| Ⅱ | 223 | 169 | 54 |  |
| Ⅲ | 244 | 133 | 111 |  |
| Ⅳ | 168 | 9 | 159 |  |
| NA | 63 | 39 | 24 |  |
| Histology |  |  |  | **P<0.001** |
| Astrocytoma, anaplastic | 129 | 53 | 76 |  |
| Astrocytoma, NOS | 66 | 41 | 25 |  |
| GBM | 168 | 9 | 159 |  |
| Mixed glioma | 132 | 89 | 43 |  |
| Oligodendroglioma, anaplastic | 82 | 60 | 22 |  |
| Oligodendroglioma, NOS | 118 | 97 | 21 |  |
| NA | 3 | 1 | 2 |  |
| TCGA_subtypes |  |  |  | 0.113 |
| Classical | 39 | 0 | 39 |  |
| Mesenchymal | 51 | 2 | 49 |  |
| Neural | 25 | 3 | 22 |  |
| Proneural | 38 | 4 | 34 |  |
| NA | 545 | 341 | 204 |  |
| KPS |  |  |  | 0.483 |
| <80 | 43 | 25 | 18 |  |
| ≥80 | 240 | 153 | 87 |  |
| NA | 415 | 172 | 243 |  |
| Treatment_or_therapy |  |  |  | **P<0.001** |
| Negative | 205 | 149 | 56 |  |
| Positive | 449 | 182 | 267 |  |
| NA | 44 | 19 | 25 |  |

Note: TCGA, The Cancer Genome Atlas; WHO, World Health Organization; GBM, glioblastoma multiforme; KPS, Karnofsky performance score; NA, not analyze.

**Supplemental Table 7** Cox regression analysis of *SYDE1* expression as a survival predictor of gliomas in CGGA mRNA-array _325.

| Parameter | Univariate analysis | | |  | Multivariate analysis | | |
| --- | --- | --- | --- | --- | --- | --- | --- |
|  | P | HR | 95%CI |  | P | HR | 95%CI |
| Age *(≥45 vs. <45 years)* | P<0.001 | 1.03 | 1.02-1.04 |  | 0.034 | 1.02 | 1.00-1.03 |
| Gender *(Male vs. Female)* | 0.613 | 0.93 | 0.71-1.23 |  | NA | NA | NA |
| WHO grade *(II vs. III vs. IV)* | P<0.001 | 2.74 | 2.28-3.30 |  | P<0.001 | 1.96 | 1.53-2.53 |
| PRS_type  *(Primary vs. Recurrent vs. Secondary)* | P<0.001 | 2.12 | 1.75-2.å57 |  | 0.009 | 2.57 | 1.20-3.57 |
| Histology  *(A vs. AA vs. AO vs. AOA vs. GBM vs. O vs. OA vs. rA vs. rAA vs. rAO vs. rAOA vs. rGBM vs. rOA vs. sGBM)* | P<0.001 | 1.12 | 1.08-1.16 |  | 0.460 | 0.96 | 0.87-1.06 |
| Radio_status *(Positive vs. Negative)* | P<0.01 | 0.54 | 0.36-0.74 |  | 0.153 | 0.75 | 0.50-1.11 |
| Chemo_status *(Positive vs. Negative)* | 0.0004 | 1.55 | 1.15-2.08 |  | 0.039 | 0.70 | 0.50-0.98 |
| IDH_mutation_status  *(Mutant vs. Wildtype)* | P<0. 01 | 0.38 | 0.29-0.51 |  | 0.809 | 0.95 | 0.64-1.41 |
| 1p19q_codeletion_status  *(Codel vs. Non-codel)* | P<0.001 | 0.17 | 0.10-0.28 |  | P<0.001 | 0.76 | 0.20-0.64 |

**Supplemental Table 8** Cox regression analysis of *SYDE1* expression as a survival predictor of gliomas in CGGA mRNA-array _693.

| Parameter | Univariate analysis | | |  | Multivariate analysis | | |
| --- | --- | --- | --- | --- | --- | --- | --- |
|  | P | HR | 95%CI |  | P | HR | 95%CI |
| Age *(≥45 vs. <45 years)* | P<0.001 | 1.03 | 1.02-1.04 |  | 0.068 | 1.01 | 1.00-1.02 |
| Gender *(Male vs. Female)* | 0.836 | 1.02 | 0.82-1.28 |  | NA | NA | NA |
| WHO grade *(II vs. III vs. IV)* | P<0.001 | 2.92 | 2.48-3.45 |  | P<0.001 | 2.29 | 1.80-2.92 |
| PRS_type *(Primary vs. Recurrent)* | P<0.001 | 1.97 | 1.58-2.46 |  | 0.002 | 2.88 | 1.48-5.58 |
| Histology *(A vs. AA vs. AO vs. AOA vs. GBM vs. O vs. OA vs. rA vs. rAA vs. rAO vs. rAOA vs. rGBM vs. rOA vs. sGBM)* | P<0.001 | 1.09 | 1.05-1.12 |  | 0.465 | 0.97 | 0.88-1.06 |
| Radio_status *(Positive vs. Negative)* | 0.019 | 1.49 | 1.07-2.08 |  | 0.985 | 1.00 | 0.67-1.49 |
| Chemo_status *(Positive vs. Negative)* | 0.004 | 1.53 | 1.14-2.04 |  | 0.093 | 0.73 | 0.50-1.05 |
| IDH_mutation_status  *(Mutant vs. Wildtype)* | P<0.001 | 3.50 | 2.77-4.42 |  | P<0.001 | 1.70 | 1.26-2.31 |
| 1p19q_codeletion_status  *(Codel vs. Non-codel)* | P<0.001 | 3.70 | 2.56-5.35 |  | 0.005 | 1.87 | 1.21-2.89 |
| *SYDE1* expression *(High vs. low)* | P<0.001 | 1.73 | 1.55-1.93 |  | 0.002 | 1.25 | 1.08-1.43 |

Note: CGGA, the Chinese Glioma Genome Altas; WHO, World Health Organization; A, astrocytomas; AA, anaplastic astrocytomas; AO, anaplastic oligodendrogliomas; AOA, anaplastic oligoastrocytomas; GBM, glioblastoma multiforme; O, oligodendrogliomas; OA, oligoastrocytomas; rA, recurrent astrocytomas; rAA, recurrent anaplastic astrocytomas; rAO, recurrent anaplastic oligodendrogliomas; rAOA, recurrent anaplastic oligoastrocytomas; rGBM, recurrent glioblastoma multiforme; rO, recurrent oligodendrogliomas; rOA, recurrent oligoastrocytomas; NA, not analyze.

**Supplemental Table 9** Cox regression analysis of *SYDE1* expression as a survival predictor of gliomas in TCGA_glioma.

| Parameter | Univariate analysis | | |  | Multivariate analysis | | |
| --- | --- | --- | --- | --- | --- | --- | --- |
|  | P | HR | 95%CI |  | P | HR | 95%CI |
| Age *(≥45 vs. <45 years)* | P<0.001 | 1.06 | 1.05-1.07 |  | P<0.001 | 1.05 | 1.03-1.07 |
| Gender *(Male vs. Female)* | 0.047 | 1.28 | 1.00-1.63 |  | 0.692 | 1.09 | 0.70-1.70 |
| Race *(Asian vs. Black or african American vs. White)* | 0.749 | 0.94 | 0.64-1.37 |  | NA | NA | NA |
| WHO grade *(II vs. III vs. IV)* | P<0.001 | 4.50 | 3.69-5.49 |  | P<0.001 | 2.84 | 1.69-4.78 |
| Histology  *(Astrocytoma, anaplastic vs. Astrocytoma, NOS vs. GBM vs. Mixed glioma vs. Oligodendroglioma, anaplastic vs. Oligodendroglioma, NOS)* | 0.993 | 1.00 | 0.92-1.08 |  | 0.993 | NA | NA |
| TCGA_subtypes *(Classical vs. Mesenchymal vs. Neural vs. Proneural)* | 0.688 | 0.97 | 0.83-1.13 |  | NA | NA | NA |
| KPS *(≥80 vs. <80 scores)* | 0.044 | 0.57 | 0.33-0.99 |  | 0.266 | 0.71 | 0.39-1.29 |
| Treatment_or_therapy *(Positive vs. Negative)* | 0.085 | 0.79 | 0.61-1.03 |  | NA | NA | NA |
| *SYDE1* expression *(High vs. low)* | P<0.001 | 2.85 | 2.45-3.32 |  | P<0.001 | 2.04 | 1.47-2.83 |

Note: TCGA, The Cancer Genome Atlas; WHO, World Health Organization; GBM, glioblastoma multiforme; KPS, Karnofsky performance score; NA, not analyze.

**Supplemental Table 10** Cox regression analysis of *SYDE1* expression as a survival predictor of LGGs in TCGA_LGG.

| Parameter | Univariate analysis | | |  | Multivariate analysis | | |
| --- | --- | --- | --- | --- | --- | --- | --- |
|  | P | HR | 95%CI |  | P | HR | 95%CI |
| Age *(≥45 vs. <45 years)* | P<0.001 | 1.05 | 1.04-1.07 |  | P<0.001 | 1.05 | 1.03-1.07 |
| Gender *(Male vs. Female)* | 0.423 | 1.15 | 0.82-1.61 |  | NA | NA | NA |
| Race *(Asian vs. Black or african American vs. White)* | 0.844 | 1.07 | 0.55-2.07 |  | NA | NA | NA |
| WHO grade *(II vs. III)* | P<0.001 | 3.05 | 2.04-4.56 |  | 0.001 | 2.57 | 1.45-4.53 |
| Histology  *(Astrocytoma, anaplastic vs. Astrocytoma, NOS vs. Mixed glioma vs. Oligodendroglioma, anaplastic vs. Oligodendroglioma, NOS)* | P<0.001 | 0.82 | 0.75-0.90 |  | 0.227 | 0.912 | 0.78-1.06 |
| KPS *(≥80 vs. <80 scores)* | 0.044 | 0.57 | 0.33-0.99 |  | 0.221 | 0.68 | 0.37-1.26 |
| Treatment_or_therapy *(Positive vs. Negative)* | 0.002 | 1.96 | 1.28-3.01 |  | 0.838 | 0.94 | 0.51-1.72 |
| *SYDE1* expression (High vs. low) | P<0.001 | 2.27 | 1.79-2.89 |  | P<0.001 | 1.91 | 1.32-2.77 |

Note: LGG, low-grade glioma; TCGA, The Cancer Genome Atlas; WHO, World Health Organization; GBM, glioblastoma multiforme; KPS, Karnofsky performance score; NA, not analyze.
